# Comprehensive analysis of alfa defensin expression and prognosis in human colorectal cancer

**DOI:** 10.3389/fonc.2022.974654

**Published:** 2023-01-10

**Authors:** Xinliang Zhao, Mengnan Lu, Zhigang Liu, Mingming Zhang, Hongmei Yuan, Zhaoqiang Dan, Daihua Wang, Bingbing Ma, Yanqi Yang, Funing Yang, Ruifang Sun, Lin Li, Chengxue Dang

**Affiliations:** ^1^ Department of Surgical Oncology, The First Affiliated Hospital of Xi’an Jiaotong University, Xi’an, Shaanxi, China; ^2^ Department of General Surgery, 521 Hospital of Norinco Group, Xi’an, Shaanxi, China; ^3^ Department of Pathology, School of Basic Medical Sciences, Health Science Center, Xi’an Jiaotong University, Xi’an, Shaanxi, China; ^4^ Department of Pediatrics, The Second Affiliated Hospital of Xi’an Jiaotong University, Xi’an, Shaanxi, China; ^5^ Department of Thoracic Surgery, Shaanxi Provincial Tumor Hospital, Xi’an, Shaanxi, China; ^6^ Department of Medical Section, 521 Hospital of Norinco Group, Xi’an, Shaanxi, China; ^7^ Department of Cardio-Thoracic Surgery, 521 Hospital of Norinco Group, Xi’an, Shaanxi, China; ^8^ Department of Clinical Laboratory, Shaanxi Provincial Tumor Hospital, Xi’an, Shaanxi, China

**Keywords:** defensin, colorectal cancer, prognosis, expression, biomarker

## Abstract

**Introduction:**

Colorectal cancer (CRC) is a serious threat to human health. Screening new biomarkers can provide basis for improving the prognosis and individualized treatment of CRC. Although some members of the defensin family were found increased in pancreatic cancer and CRC, their exact function and clinical significance remain unclear.

**Methods:**

In this study, the expression, correlation, mutation, and functional enrichment of several defensin family members in pancreatic cancer and CRC were analyzed using tumor public databases and verified in several patients.

**Results:**

Results showed no significant correlation between the expression levels of DEFA1-4 and CRC. The expression levels of DEFA5 and DEFA6 significantly increased in CRC tissues compared with those in normal tissues. DEFA5 may be associated with better prognosis of CRC, while DEFA6 may be associated with poor prognosis. Immunohistochemistry (IHC) experiments showed that the expression of DEFA6 was significantly higher in adenoma than in normal mucosa and slightly higher in carcinoma than in normal mucosa. The Kyoto Encyclopedia of Genes and Genomes (KEGG) analysis found that DEFAs were closely related to hsa05202: transcriptional misregulation in cancer and Hsa04015: Rap1 signaling pathway. DEFA5 may be a stable and good prognostic marker, and DEFA6 may be a poor prognostic marker in CRC of metastasis.

**Conclusion:**

Overall, DEFA5 and DEFA6 have a certain degree of sensitivity and specificity in predicting CRC.

## Introduction

The incidence rate of colorectal cancer (CRC) ranks third in new cancer cases worldwide, and the mortality rate of CRC ranks second among all cancers. CRC is a common malignant tumor in the digestive system, and the typical lesion sites are the colon, rectum, and appendix (1). The main treatment methods of CRC are surgery, chemotherapy, radiotherapy, and targeted therapy. However, 40% of confirmed cases of CRC will die of recurrence or metastasis. The gene tests of Kirsten Rat Sarcoma Viral Oncogene (KRAS) (2) and B-Raf Proto-Oncogene, Serine/Threonine Kinase (BRAF) have been used as routine basis for adjuvant therapy, and the detection of wild type or mutant can guide the selection of targeted drugs for initial unresectable CRC. Although some tumor makers, such as carcinoembryonic antigen (CEA), have been used for screening and predicting the recurrence of CRC, few sensitive and specific detection indices are currently available for CRC (3). Therefore, new biomarkers should be explored to improve the prognosis evaluation and individualized treatment for CRC.

Defensins belong to antimicrobial and cytotoxic peptides that are involved in host defense. Apart from their role in the innate immune systems, many studies have indicated that defensins are expressed and involved in some kinds of cancer, including CRC, breast cancer, and kidney cancer. Defensins are divided into three different subfamilies: α-defensin, β-defensin, and θ-defensin. In many mammals, α-defensin is one of the potent antimicrobial peptides expressed on the mucosal surface. It mainly exists in the epithelial cells of the intestine, respiratory tract, urinary tract, and vagina and is abundant in the granules of neutrophils. α-Defensin contains five members, DEFA1 (also known as DEFA2, HNP-1, HNP-2), DEFA3 (also known as HNP-3), DEFA4 (also known as HNP-4), DEFA5, and DEFA6 (4). The human normalized signal intensity Protein Atlas (HPA, https://www.proteinatlas.org/) shows the distribution of DEFAs expression in different human tissues. We found that DEFA1 and DEFA3 are mainly expressed in bone marrow and spleen, DEFA4 is mainly expressed in bone marrow, lymph node, and spleen, and DEFA5 and 6 are mainly expressed in duodenum and small intestine. In HPA, the expression of DEFA6 in 11 tissues shows nine without the detected protein, one with medium expression, and one with low expression. DEFA5 and DEFA6, also known as intestinal defensins, are produced by Paneth cells (5). The antimicrobial activity of these peptides shows inhibition to various microorganisms, including bacteria (6), fungi, viruses, and protozoan parasites (7).

The relationship between DEFAs and malignant tumors has been partially reported. The plasma levels of DEFA1-3 in patients with bladder cancer are parallel to the progression and pathological stage of malignant tumor, suggesting that DEFA1–3 can promote bladder cancer invasion and could be potential indicators of disease progression (8). DEFA1, DEFA2, and DEFA3 are expressed in renal cell carcinoma cells and may directly affect tumor proliferation (9). α-Defensins (DEFA1–3) are overexpressed in patients with breast cancer with complete remission after paclitaxel therapy (10). The gene expression levels of DEFA1, DEFA3, and DEFA4 increased significantly in adenocarcinoma, adenoidcystic carcinoma, and mucoepidermoid carcinoma compared with healthy salivary gland tissues (11).Tissue expression analysis showed that DEFA1–3 in gastric cancer increased 10 times compared with that in adjacent normal mucosa (P=0.001) (12). DEFA5 had inhibitory effect on the growth of gastric cancer cells and might play a potential antitumor role in gastric cancer (13).

DEFAs have been found to be closely related to CRC. DEFA1-3 were found increased in the serum and tissues in CRC. The expression levels of DEFA1-3 in the serum and tissues of colorectal adenomas and cancers are higher than that in the normal epithelium. The average sensitivity levels of DEFA1–3 in patients’ serum to diagnose CRC was 69%, and the specificity was 100% (14–16). The expression levels of DEFA1–3 were correlated to lymphatic or hepaticmetastasis.HNP1-3 were expressed in tumor cells rather than in neutrophils (17).DEFA5 were found inhibit progression of CRC and maybe a favorable prognostic factors. The protein expression of DEFA5 in colon tumors significantly increased (18). Some scholars detected the expression of DEFAs in colon cancer, adenoma, and surrounding normal tissues. DEFA5 and DEFA6 are considered as key factors in colon adenoma formation (19). DEFA5 showed strong killing effect on colon cancer cells and did not affect normal host cells (20). DEFA5 showed an inhibitory effect in colon cancer cell growth and may serve as a potential tumor suppressor in colon cancer (21). DEFA6 has different structures and lacks antibacterial properties (22). DEFA6 were found to promote the occurrence of colon adenoma and colon cancer. DEFA6 is not expressed in any other tissues except the gastrointestinal tract (stomach and colon). Expression microarray data analysis obtained from 283 tumors and normal tissues showed that DEFA6 was maximally expressed in colon cancer (23). Other studies also found significantly increased DEFA6 expression in colon adenoma and cancer (24). By inhibiting the function of DEFA6 by shRNA, DEFA6 promoted the proliferation, migration, invasion, and colony forming ability of CRC cells (25). A previous work that analyzed 352 patients with CRC reported that the high expression of DEFA6 was associated with the poor survival rate of patients and could be an independent prognostic marker of CRC (25).

The expression of DEFA family genes has been partially reported in human CRC. However, the comprehensive expression, mutation, enrichment, and immune infiltration of whole family members in the tumor database are unclear. In the present study, we used bioinformatics combined with tumor-related database and existing published literature to analyze the expression and correlation of the whole DEFA family members with pancreatic cancer and CRC in detail and determine their potential function and prognostic value.

## Materials and methods

### Materials

Data were downloaded at UCSC XENA (https://xenabrowser.net/datapages/) in RNAseq data in TPM format for TCGA and GTEx processed uniformly by the Toil process (26). The extracted COAD (colon cancer) of TCGA and the corresponding normal tissue data in GTEx, including 308 normal, 41 para-carcinoma tissue, and 480 tumors. Among them, gender group is: female 226, male 252; age group is:<=65 194,>65 284; T stage is: T1 11, T2 = 83, T3 = 323, T4 = 60; N stage is: N0 = 284, N1 = 108, N2 = 86; M stage is: M0 = 349, M1 = 66.

## Publicly available database

### Oncomine analysis

As a large tumor gene chip database, Oncomine gene expression array datasets (https://www.oncomine.org/resource/login.html, an online cancer microarray database) collected sample data of 86733 tumors and normal tissues from nearly 715 datasets. We selected DEFA1-6 as the target gene. Firstly, we analyzed the transcription levels of DEFAs in different cancers. Further, we explored the difference in the expression of DEFAs between different tumor types and normal tissues in CRC. The mRNA expression of DEFAs in clinical cancer specimens was compared with that in normal controls by using Student’s t test to generate a P value. Cut-off P value and fold change were defined as 0.01 and 2, respectively.

### Gene expression profiles of colorectal cancer

We downloaded gene profiles (GSE15781) from the Gene Expression Omnibus (GEO) database (https://www.ncbi.nlm.nih.gov/geo/). This study examined the gene expression profile in normal (n=10) and adenocarcinomas (n=13) by gene expression microarray. We further analyzed the differential gene expression of rectal genes, and details on the samples can be found in the original article [PMID: 19969511]. GSE15781 was downloaded from the GEO database through the GEOquery package. If probes corresponding to multiple molecules were removed, and if probes corresponding to the same molecule were encountered, only the probe with the largest signal value was retained. Statistical analysis and visualization were performed using the R packages “GEOquery” (27), “limma” (28), “ComplexHeatmap” (29), and “ggplot2.” Differentially expressed genes (DEGs) were identified based on |log2FC|>1 and adjusted p-values<0.05. Further analyses for DEGs were performed using the R packages “org.Hs.eg.db” and “clusterProfiler.”

### GEPIA2 dataset

GEPIA2 (Gene Expression Profiling Interactive Analysis) dataset (GEPIA 2 (cancer-pku.cn)), is an updated version of GEPIA for analysis of the RNA sequencing expression data of 9,736 tumors and 8,587 normal samples from the TCGA and the Genotype Tissue Expression (GTEx) projects by using a standard processing pipeline. GEPIA2 provides customizable functions such as tumor/normal differential expression analysis, profiling according to cancer types or pathological stages, patient survival analysis, similar gene detection, correlation analysis, and dimensionality reduction analysis (30). We used GEPIA2 to investigate the difference in the expression of DEFAs in colorectal cancer tissues and surrounding normal tissues, and in different stages of colorectal cancer, so as to find out the influence of DEFAs in the occurrence and development of colorectal cancer.

### HPA database and Immunohistochemistry

HPA (The Human Protein Atlas https://www.proteinatlas.org/) database aim to map all the human proteins in cells, tissues, and organs using an integration of various omics technologies. We showed the protein expression of DEFAs in colorectal cancer through HPA database. To verify the results, we performed immunohistochemistry in 5 patients with colorectal cancer. Tumor sections (3mm) were incubated with commercial rabbit polyclonal antibodies against DEFA6 at 1/100 dilution overnight at 4°C. The sections were conjugated with horseradish peroxidase (HRP) antibody at room temperature for 2h and then covered by 3,3-diaminobenzidine (DAB). Slides were mounted with neutral resin seal. All fields were observed under light microscopy. Control experiments without primary antibody demonstrated that the signals observed were specific.

### Clinical data analysis

In order to understand DEFAs and prognosis and the ability to predict prognosis, using data in Materials part, statistical analysis and visualization of prognosis were performed in R (v. 3.6.3). R packages involved: “survival” (v. 3.2-10) for statistical analysis of survival data, “survminer” (v. 0.4.9) for visualization. ROC statistical analysis and visualization were performed in R with “pROC” (v. 1.17.0.1) and “ggplot2”.

### Cancer genome atlas data and cBioPortal

The Cancer Genome Atlas (TCGA) has sequencing and pathological data on 30 different cancers. TCGA Pan Cancer Atlas Studies including 10953 patients, was selected for further analyses of DEFAs by using cBioPortal (http://www.cbioportal.org/). Based on cBioPortal, we analyzed the mutation rate of DEFAs in CRC, the mutation rates of DEFA6 in pancreatic cancer, and the correlations between the mRNA expression of DEFAs in patients with CRC (Pearson correlation coefficient). Further, we searched for 100 related genes of DEFA5 and DEFA6 in colorectal cancer, and speculated the possible role of them in the carcinogenesis of colorectal cancer by analyzing the role and pathway of the top genes. The genomic profiles included mutations, putative copy number alterations (CNAs) from genomic identification of significant targets in cancer (GISTIC), mRNA expression Z scores (RNA-seq v.2 RSEM), and protein expression Z scores (reverse-phase protein array, RPPA). Co-expression and network were calculated according to the cBioPortal’s online instructions (31).

### Protein–protein interaction network analysis

Based on the related genes of DEFAs in CRC, PPI can be used to help us find the most closely related expressed proteins with DEFAs. A PPI network was generated using the STRING v11.5 database (STRING, https://string-db.org). The interaction network was generated with a confidence score of 0.15 and “Active interaction sources” based on molecular biological experiments (32).

### Gene enrichment analysis

The enrichment analysis of Gene Ontology (GO) was performed to enrich differentially expressed genes (DEGs) according to biological process (BP), cellular component (CC), and molecular function (MF). The enrichment analysis of Kyoto Encyclopedia of Genes and Genomes (KEGG) was carried out to identify and confirm related signal pathways of DEGs. We used the above tools to understand the biological process involved in colorectal cancer by DEFAs. GO and KEGG enrichment was made by R package modules of “org.Hs.eg.db” (v.3.11.4) and “clusterProfiler” (v.3.17.3). Functional pathway visualization based on sequencing data was conducted by R package “pathview” (v.1.29.1). The statistical package of R software (R Foundation for Statistical Computing, Vienna, Austria. https://www.R-project.org/) was used for correlation analysis with the RNA-seq data database in the COAD (colon cancer) project from the TCGA database.

### TIMER dataset

The relationship between DEFAs and immune cell infiltration was shown in CRC. The TIMER web (https://cistrome.shinyapps.io/timer/) server is a comprehensive resource for systematic analysis of immune infiltrates across diverse cancer types. The abundance of six immune infiltrates (B cells, CD4+ T cells, CD8+ T cells, neutrophils, macrophages, and dendritic cells) was estimated by TIMER algorithm. TIMER web server allows users to input function-specific parameters, with resulting figures dynamically displayed to conveniently access the immunological, clinical, and genomic features of the tumor (33).

### Ethics statement

This study was approved by the Academic Committee of Xi’an Jiaotong University and conducted according to the principles expressed in the Declaration of Helsinki. All the datasets were retrieved from the published literature, so it was confirmed that all written informed consent was obtained.

## Results

### Transcriptional expression levels of DEFAs in patients with tumors

**Figure 1:**
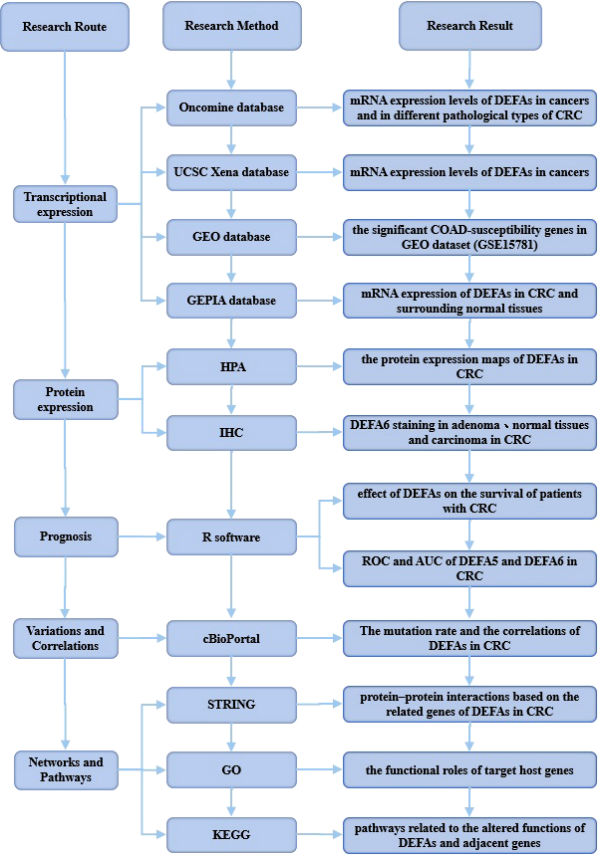
Flow diagram of the data collection and method implementation in this work.

**Figure 2:**
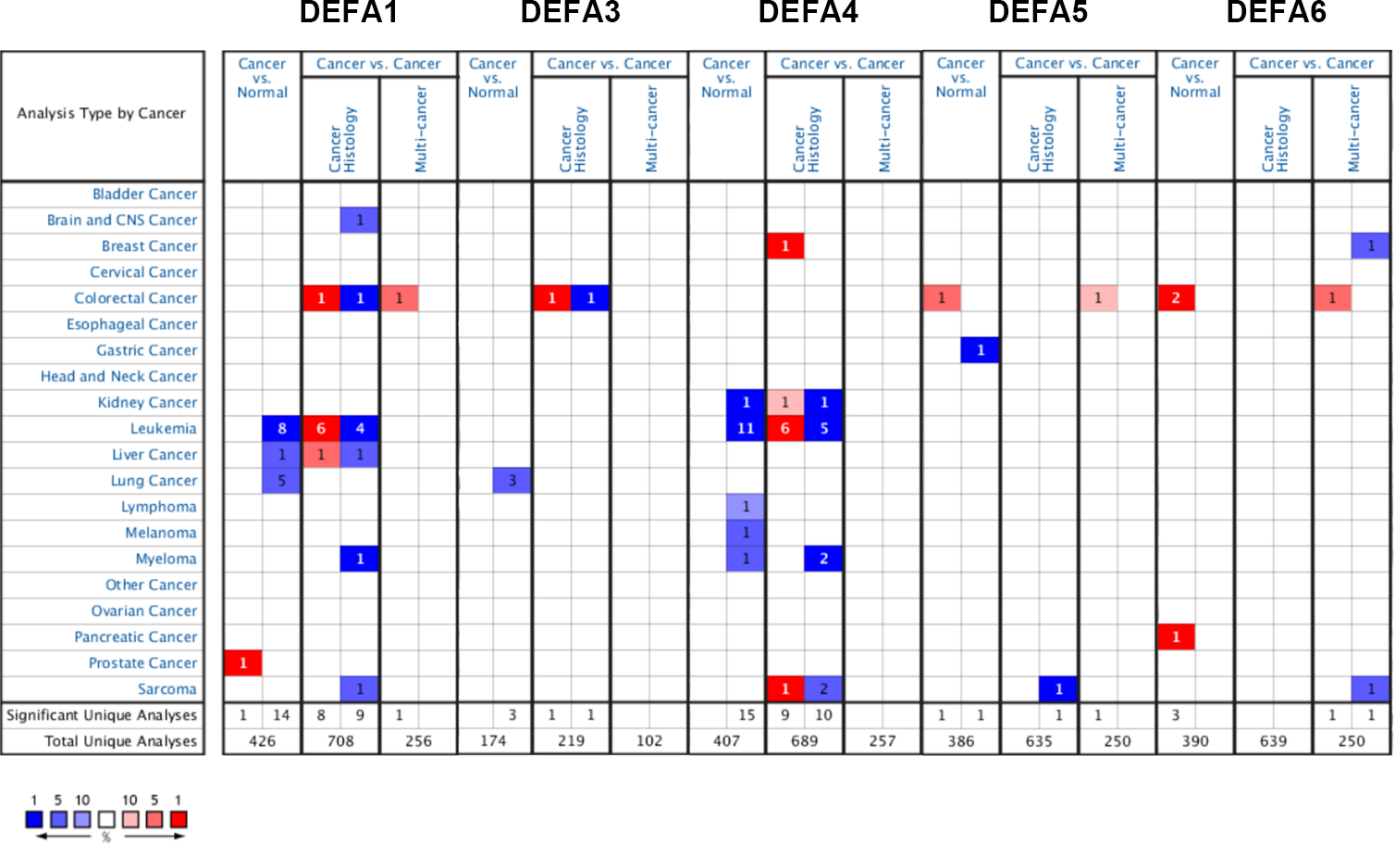
The transcription levels of DEFA factors in different types of cancers (Oncomine).

We made a flow chart to show our process (**Figure 1**). We compared the expression levels of DEFAs in cancers with those in normal samples by using the Oncomine database (https://www.oncomine.org) (**Figure 2**). In 426 studies on DEFA1, one study showed high expression and 14 studies showed low expression. In 174 studies on DEFA3, three studies showed low expression. In 407 studies on DEFA4, 15 studies showed low expression. In 386 studies on DEFA5, two studies showed high expression in CRC and low expression in gastric cancer. In 390 studies on DEFA6, three studies showed high expression and 2 of them were in CRC.

**Figure 3:**
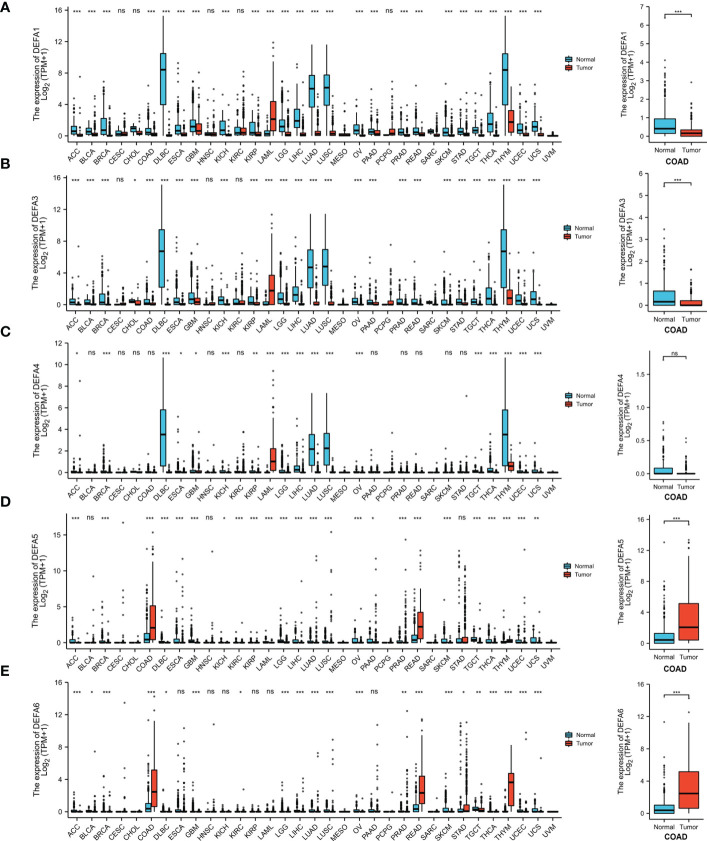
The transcription levels of DEFA factors in different types of cancers (UCSC XENA) **(A)**. The expression level of DEFA1 in in different types of cancers; **(B)**. The expression level of DEFA3 in in different types of cancers; **(C)**. The expression level of DEFA4 in in different types of cancers; **(D)**. The expression level of DEFA5 in in different types of cancers; **(E)**. The expression level of DEFA6 in in different types of cancers.

To further confirm these results, Using the UCSC Xena database (https://xenabrowser.net/datapages/), Data were downloaded in RNAseq data in TPM format for TCGA and GTEx processed uniformly by the Toil process (26). The extracted Pan-Cancer of TCGA and the corresponding normal tissue data in GTEx were visualized using R package “ggplot2” (v.3.3.3) (**Figure 3**). The mRNA expression levels of DEFA1, DEFA3, and DEFA4 in tumor tissues were generally lower than those in normal tissues but significantly higher in tumor cells of acute myeloid leukemia than those in normal tissues. The expression of DEFA5 in tumor tissues was generally lower than that in normal tissues; however, the expression of DEFA5 in colon cancer and rectal adenocarcinoma cells was significantly higher than that in normal tissues. The expression of DEFA6 in colon cancer, rectal adenocarcinoma, thymic cancer, and gastric cancer was significantly higher than that in normal tissues. The above results showed that in the DEFA family, in cancers, DEFA5 and DEFA6 were closely related to colorectal cancer, and their expressions in CRC were higher than those in normal tissues.

Furthermore, we compared the transcriptional levels of DEFAs in different pathological types of CRC with those in normal samples by using the Oncomine databases (**Table 1**). In CRC, we found the mRNA expression levels of DEFAs were upregulated in different levels. Compared with that in normal tissues, the fold change of DEFA1 was 1.235 in colon cancer (Zou Colon Dataset) (34), 1.261 in CRC (Skrzypczak Colorectal Dataset) (35), 1.270 in colonic mucinous adenocarcinoma (Kaiser Colo Dataset) (36), 1.171 in colonic adenocarcinoma (Kaiser Colo Dataset) (36), and 1.442 in CRC (TCGA Colorectal Statistics). Compared with normal tissues, the fold change of DEFA3 in colonic mucinous adenocarcinoma was 1.986 (TCGA Colorectal Statistics). The fold change of DEFA4 in colorectal adenocarcinoma was 1.105 (Skrzypczak Colorectal Statistics) (35). The fold changes of DEFA5 in colorectal adenocarcinoma were15.963 (Skrzypczak Colorectal Statistics) (35),2.740 (TCGA Colorectal Statistics), and 2.440 (Hong Colorectal Statistics) (37). In the Skrzypczak Colorectal Statistics (35), the fold changes of DEFA6 were 24.571 in colorectal adenocarcinoma and 3.953 in CRC. In TCGA Colorectal Statistics, the fold changes of DEFA6 were 2.780 in colonic mucinous adenocarcinoma and 2.288 in colonic adenocarcinoma. InKaiser Colon Statistics (36), the fold change of DEFA6 in colonic adenocarcinoma was 2.345. In the Sabates-Bellver Colon Statistics (38), the fold change of DEFA5 was 15.604 and that of DEFA6 was 72.141 in colon adenoma. The data set is shown in **Table 2**. The above results suggest that there is no significant difference between DEFA5,6 in colon, rectum, adenoma and non-adenoma.

**Table 1:** The fold changes of DEFAs expression in transcription levels between varied types of CRC and normal colorectal tissues (Oncomine Database).

	Type of Colon Cancer	Fold Change (Colon Cancer vs Normal Colon Tissue)	p Value	t Test	Source
DEFA1	Colon Carcinoma	1.235	***0.013***	2.501	Zou Colon Statistics
	Colorectal Carcinoma	1.261	***1.80E-04***	3.794	Skrzypczak Colorectal Statistics
	Colon Adenocarcinoma	-1.429	0.97	-1.948	Notterman Colon Statistics
	Colon Mucinous Adenocarcinoma	1.27	***0.009***	2.765	Kaiser Colon Statistics
	Colon Adenocarcinoma	1.171	***0.031***	2.433	Kaiser Colon Statistics
	Colorectal Adenoma Epithelia	-1.051	0.737	-0.638	Gaspar Colon Statistics
	Colon Mucinous Adenocarcinoma	1.442	0.073	1.484	TCGA Colorectal Statistics
	Colon Adenocarcinoma	-1.385	0.978	-2.115	TCGA Colorectal Statistics
	Colorectal Carcinoma	1.002	0.495	0.012	Hong Colorectal Statistics
	Colon Adenoma	1.066	0.397	0.262	Sabates-Bellver Colon Statistics
DEFA3	Colon Mucinous Adenocarcinoma	1.135	0.082	1.826	Kurashina Colon Statistics
	Colon Adenocarcinoma	-1.067	0.999	-3.254	Kurashina Colon Statistics
	Colon Mucinous Adenocarcinoma	1.986	***0.006***	2.68	TCGA Colorectal Statistics
	Colon Adenocarcinoma	-1.058	0.624	-0.32	TCGA Colorectal Statistics
DEFA4	Colon Mucinous Adenocarcinoma	1.134	0.084	1.805	Kurashina Colon Statistics
	Colon Adenocarcinoma	-1.074	1	-3.526	Kurashina Colon Statistics
	Colorectal Adenoma Epithelia	1.045	0.107	1.256	Gaspar Colon Statistics
	Colorectal Adenocarcinoma	1.105	***0.004***	2.717	Skrzypczak Colorectal Statistics
	Colorectal Carcinoma	1.029	0.239	0.716	Skrzypczak Colorectal Statistics
	Colon Adenocarcinoma	-1.14	0.942	-1.587	Ki Colon Statistics
	Colon Mucinous Adenocarcinoma	-1.006	0.514	-0.036	TCGA Colorectal Statistics
	Colon Adenocarcinoma	-1.113	0.779	-0.78	TCGA Colorectal Statistics
	Colon Adenocarcinoma	-1.118	0.881	-1.364	Kaiser Colon Statistics
	Colon Mucinous Adenocarcinoma	-1.111	0.866	-1.25	Kaiser Colon Statistics
	Colon Adenoma	-1.259	0.843	-1.017	Sabates-Bellver Colon Statistics
	Colorectal Carcinoma	-1.357	0.907	-1.38	Hong Colorectal Statistics
DEFA5	Colorectal Adenocarcinoma	15.963	***4.57E-10***	7.131	Skrzypczak Colorectal Statistics
	Colon Adenocarcinoma	1.334	0.323	0.463	Notterman Colon Statistics
	Colon Adenoma	15.604	***7.74E-08***	6.129	Sabates-Bellver Colon Statistics
	Colon Mucinous Adenocarcinoma	1.135	0.082	1.825	Kurashina Colon Statistics
	Colon Adenocarcinoma	-1.072	1	-3.526	Kurashina Colon Statistics
	Colon Mucinous Adenocarcinoma	2.74	***0.02***	2.127	TCGA Colorectal Statistics
	Colon Adenocarcinoma	1.745	0.066	1.544	TCGA Colorectal Statistics
	Colorectal Carcinoma	2.44	***0.014***	2.372	Hong Colorectal Statistics
	Colon Adenocarcinoma	1.651	0.207	0.889	Kaiser Colon Statistics
	Colon Mucinous Adenocarcinoma	1.666	0.229	0.777	Kaiser Colon Statistics
DEFA6	Colorectal Adenocarcinoma	24.571	***2.73E-16***	10.851	Skrzypczak Colorectal Statistics
	Colorectal Carcinoma	3.953	***2.02E-05***	4.504	Skrzypczak Colorectal Statistics
	Colon Adenoma	72.141	***2.09E-14***	10.166	Sabates-Bellver Colon Statistics
	Colon Mucinous Adenocarcinoma	1.134	0.084	1.805	Kurashina Colon Statistics
	Colon Adenocarcinoma	-1.074	1	-3.526	Kurashina Colon Statistics
	Colon Adenocarcinoma	-1.615	0.863	-1.119	Notterman Colon Statistics
	Colon Mucinous Adenocarcinoma	2.78	***0.004***	2.821	TCGA Colorectal Statistics
	Colon Adenocarcinoma	2.288	***7.77E-04***	3.415	TCGA Colorectal Statistics
	Colon Adenocarcinoma	2.345	***0.007***	2.895	Kaiser Colon Statistics
	Colon Mucinous Adenocarcinoma	1.491	0.149	1.077	Kaiser Colon Statistics

P values with statistical differences are shown in bold and italic.

**Table 2:** Statistics Source and the characteristics of the samples (Oncomine Database).

Number of Tumor	Number of Normal	Statistics Source
94	94	Zou Colon Statistics(28351398)
105	40	Skrzypczak Colorectal Statistics(20957034)
55	50	Notterman Colon Statistics(19359472)
36	40	Kaiser Colon Statistics(17615082)
6	6	Gaspar Colon Statistics(29547900)
480	41	TCGA Colorectal Statistics
70	12	Hong Colorectal Statistics(20143136)
32	32	Sabates-Bellver Colon Statistics(18171984)
10	10	Kurashina Colon Statistics(16247484)
27	25	Ki Colon Statistics(17640062)

### Relationship between the mRNA levels of DEFAs and the clinicopathological parameters of patients with CRC

**Figure 4:**
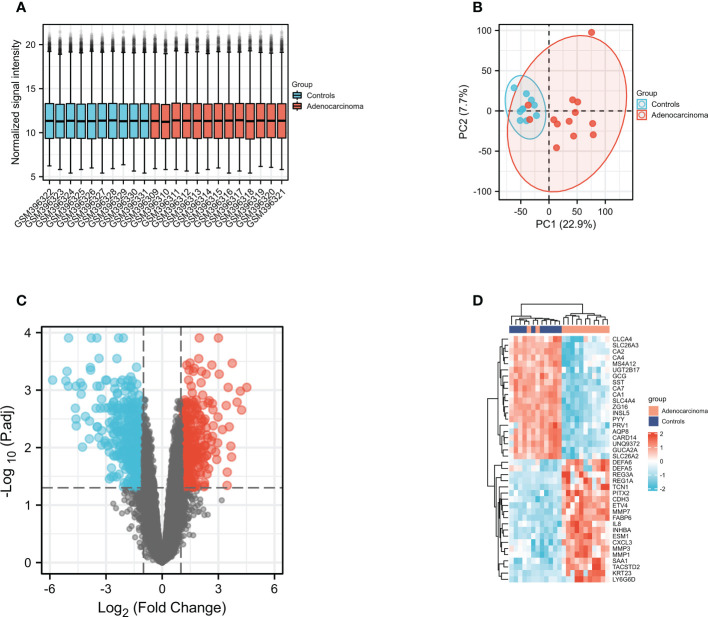
Gene expression profiles of COAD. **(A)** Normalized bar plot of the GSE715781 dataset; **(B)** PCA analysis of the GSE15781 dataset; **(C)** Vocanol plot of the GSE15781 dataset. Gene expression analysis of the GSE15781 dataset; **(D)** Heatmap of the top 40 genes in GSE15781 dataset.

To identified whether DEFAs were the significant COAD-susceptibility genes, we selected and analyzed GEO dataset (GSE15781). GSE15781 dataset were normalized and corrected (**Figure 4A**), and principal component analysis (**Figure 4B**) showed significant differences between subgroups, which suggested that a follow-up analysis of variance would be meaningful. GSE15781 identified 929 DEGs (**Figure 4C**), respectively,421 up-regulated and 508 down-regulated (|log2(FC)|>1 & p.adj<0.05). The top 40 DEGs are shown in a clustered heatmap (**Figure 4D**); the up-regulated DEGs are related to DEFA5 and DEFA6.

**Figure 5:**
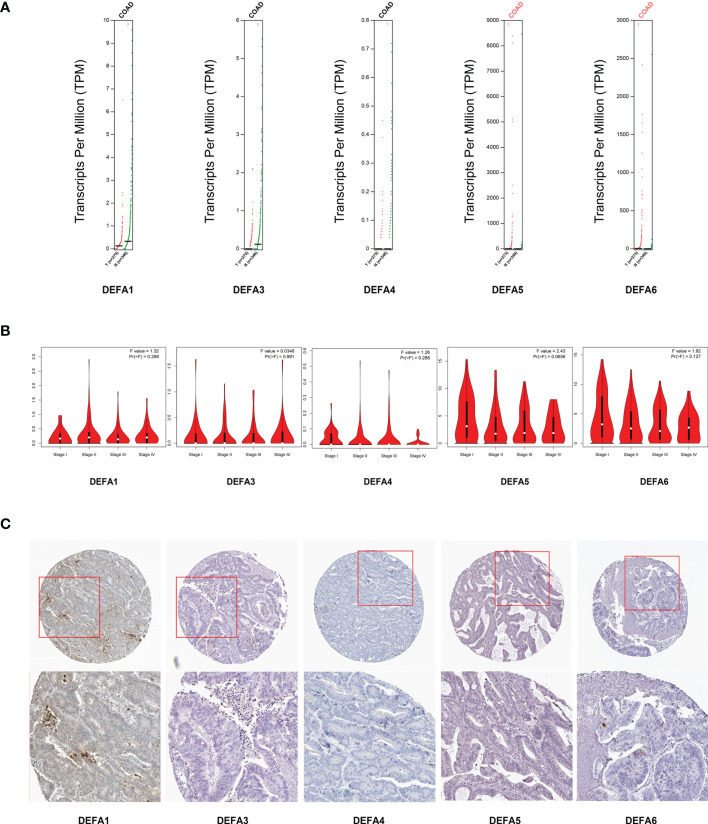
Relationship between the mRNA levels of DEFAs and the clinicopathological parameters of patients with CRC. **(A)** Transcription levels of DEFA factors in CRC (GEPIA2); **(B)** Correlation between DEFAs expression and tumor stages in CRC patients (GEPIA2); **(C)** Protein expression of DEFAs in CRC patients (HPA).

Through GEPIA2 (Gene Expression Profiling Interactive Analysis) dataset (GEPIA 2 (cancer-pku.cn)), we compared the mRNA expression of DEFAs in CRC and surrounding normal tissues (**Figure 5A**). The expression of DEFA5 and DEFA6 increased in CRC. We also analyzed the expression of DEFAs in different tumor stages of CRC. No significant difference was found between stages (**Figure 5B**). This result suggests that DEFA5 and DEFA6 may play a role in the occurrence rather than development of colorectal cancer.

**Figure 6:**
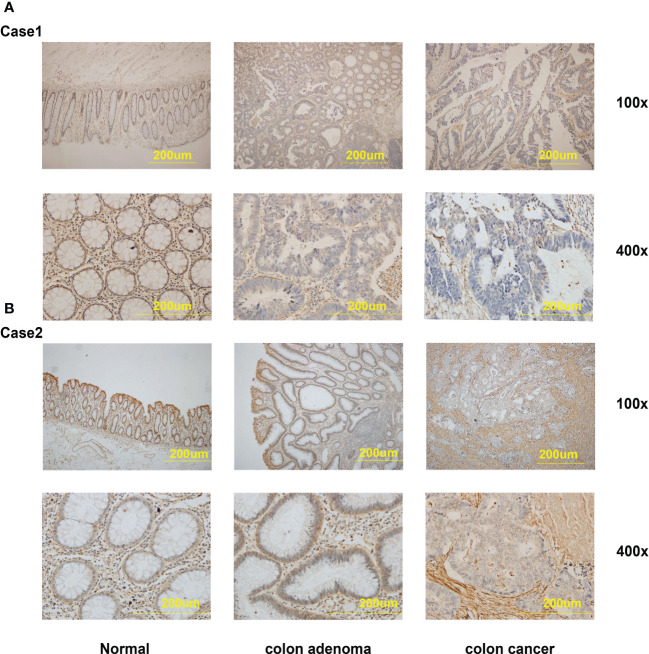
The IHC staining of DEFA6 in the same patients with normal mucosa, colon adenoma and colon carcinoma. **(A)** The IHC staining of DEFA6 in case 1 patient; **(B)** The IHC staining of DEFA6 in case 2 patient.

We further gathered the protein expression maps of DEFAs in CRC in the HPA database (https://www.proteinatlas.org/). Compared with normal tissues, no change in DEFA1 and DEFA3-5 was found between CRC and normal tissues, while the protein expression of DEFA6 increased in CRC compared with that in normal tissues (**Figure 5C**). Moreover, we performed immunohistochemical staining on five patients who carried normal colon mucosa, adenoma, and colon carcinoma at the same time. Detailed patient information is shown in **Supplementary Table S1**. Considering that the sample size was relatively small, we only showed the results of two patients(number1 and number 2 cases in **Supplementary Table S1**)as representatives. The stain was distributed in the cytoplasm, plasma membrane, and extracellular membrane. The intensity of DEFA6 staining in adenoma was obviously deeper than that in normal tissues. DEFA6 staining in carcinoma was slightly deeper than that in normal tissues (**Figures 6A, B**).

### Association of the mRNA expression of DEFA5 and DEFA6 with prognosis and survival prediction ability of patients with CRC

**Figure 7:**
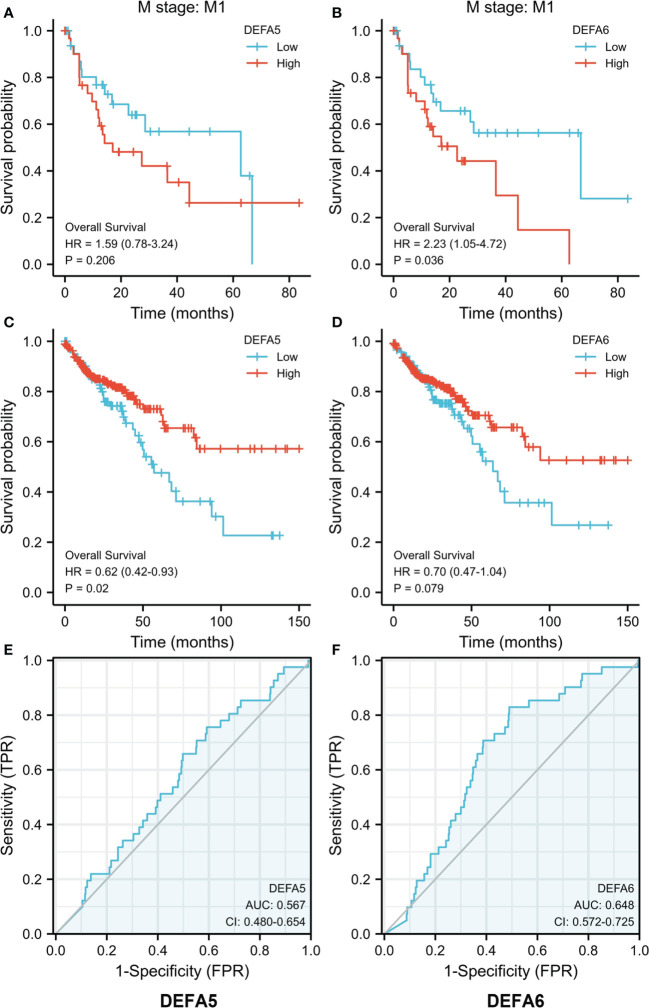
The prognostic value of DEFA5 and DEFA6 in patients with CRC. **(A)**. Correlation between DEFA5 and overall survival of patients with CRC; **(B)** Correlation between DEFA6 and overall survival of patients with CRC; **(C)** Correlation between DEFA5 and overall survival of M1 stage patients with CRC; **(D)** Correlation between DEFA6 and overall survival of M1 stage patients with CRC; **(E)** ROC Curves of DEFA5 in patients with CRC; **(F)** ROC Curves of DEFA6 in patients with CRC.

We further explored the effect of DEFAs on the survival of patients with CRC based on the data downloaded at UCSC XENA including 480 tumors by using the survminer package of R software (**Figures 7A–D**) (39). DEFA1–4 had no data or effect on the survival of CRC (data not shown). The higher expression of DEFA5 was associated with longer overall survival (OS) in patients with CRC (HR=0.62, P=0.02) (**Figure 7C**). In patients with M1 stage CRC, the higher expression of DEFA6 was associated with shorter overall survival (OS) (HR=2.23, P=0.036) (**Figure 7B**). This result suggests that the increase of DEFA5 is associated with a good prognosis of colorectal cancer, while the increase of DEFA6 is associated with a poor prognosis of advanced colorectal cancer.

By using the pROC package of R software, we analyzed the RNA seq data in Level 3 HTSeq-FPKM format in the COAD (colon cancer) project in the TCGA database (https://portal.gdc.cancer.gov/). As such, Receiver Operating Characteristic (ROC) curves were made. The Area Under Curve (AUC)of DEFA5 is 0.567, while that of DEFA6 is 0.648, suggesting that DEFA5 (**Figure 7E**) and DEFA6 (**Figure 7F**) have a certain degree of sensitivity and specificity in predicting CRC.

### Predicted functions and pathways deduced from the changes in DEFAs and their frequently altered neighbor genes in CRC

**Figure 8:**
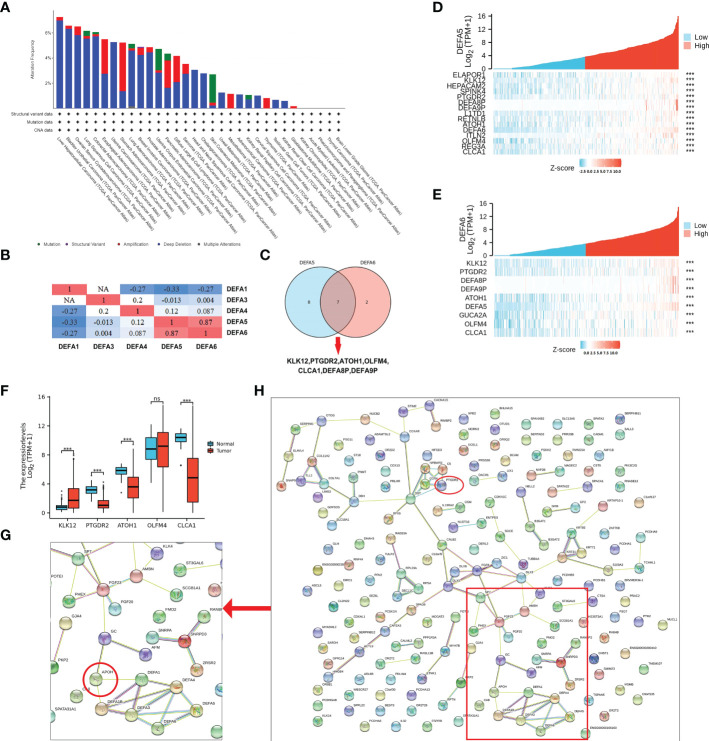
Mutation and co-expression genes of DEFAs in CRC. **(A)** Mutation rates of DEFA6 in pan-cancer; **(B)** Correlations among DEFAs; **(C)** Co-expressed gene Wayne diagram of DEFA5 and DEFA6; **(D)** Co-expression gene of DEFA5; **(E)** Different expression of Hub gene; **(F)** Co-expression gene of DEFA6; **(G)** Key proteins near DEFAs in protein protein interaction network (PPI network); **(H)** PPI network of related genes of DEFA5 and DEFA6.

We analyzed the variations, correlations, and networks of DEFAs by using the cBioPortal online tool (cBioPortal for Cancer Genomics; https://www.cbioportal.org/). The mutation rate of DEFA1 and DEFA3–6 in CRC was 7% (**Supplementary Figure S1**). In pancreatic cancer, the mutation rates of DEFA6 were analyzed using the TCGA database. The results showed that the mutation rates in hepatocellular carcinoma, bladder urothelial carcinoma, ovarian serous cystadenocarcinoma, lung squamous cell carcinoma, and colorectal adenocarcinoma were all higher than 6%; the mutation rate of DEFA6 in CRC ranked fifth in pancreatic cancer, mainly was deep deletion (**Figure 8A**). We continued to analyze the correlations between the mRNA expression of DEFAs in patients with CRC (Pearson correlation coefficient) (**Figure 8B**). The results showed significant positive correlations between DEFAs as follows: DEFA3 with DEFA6; DEFA4 with DEFA5 and DEFA6; DEFA5 with DEFA4 and DEFA6; DEFA6 with DEFA3; and DEFA4 and DEFA5. The above results suggest that the expressions of DEFA5 and DEFA6 are highly consistent.

As shown in **Supplementary Table S2**, the expression levels of DEFA5 (**Figure 8D**) had strong positive correlations with KLK12 (R=0.57), CLCA1 (R=0.49), L1TD1 (R=0.47), REG3A (R=0.46), HEPACAM2 (R=0.44), ITLN2 (R=0.43), OLFM4 (R=0.43), RETNLB (R=0.43), PTGDR2 (R=0.43), ATOH1 (R=0.42), SPINK4 (R=0.42), and ELAPOR1(R=0.41). As shown in **Supplementary Table S3**, the expression levels of DEFA6 (**Figure 8E**) had strong positive correlations with KLK12 (R=0.57), OLFM4 (R=0.45), CLCA1 (R=0.43), PTGDR2 (R=0.43), GUCA2A (R=0.43), and ATOH1 (R=0.41). We selected the co-expressed genes with the highest correlation between DEFA5 and DEFA6 (**Figure 8C**): KLK12, PTGDR2, ATOH1, OLFM4, and CLCA1, which are successively involved in tumorigenesis, inflammatory response, transcriptional regulation, inhibition of cell growth, induction of cell differentiation and apoptosis, and promotion of cell adhesion and tumor inhibition. In particular, KLK12, which is responsible for tumor tumorigenesis, is highly expressed in CRC (**Figure 8F**).

The STRING (hppt://string-db.org) online database is used to search for known proteins and predict protein–protein interactions (PPI), including direct physical interactions and indirect functional correlations between proteins. Based on the related genes of DEFAs in CRC, we constructed a network for DEFAs and the respective most frequently changed neighbor genes (**Figure 8H**). The result significantly showed that DEFAs were closely related to Apolipoprotein (APOH) (**Figure 8G**), which was related to hyperlipidemia and closely related to the incidence of CRC. Hence, DEFAs might affect the occurrence and development of CRC through the APOH pathway.

**Figure 9:**
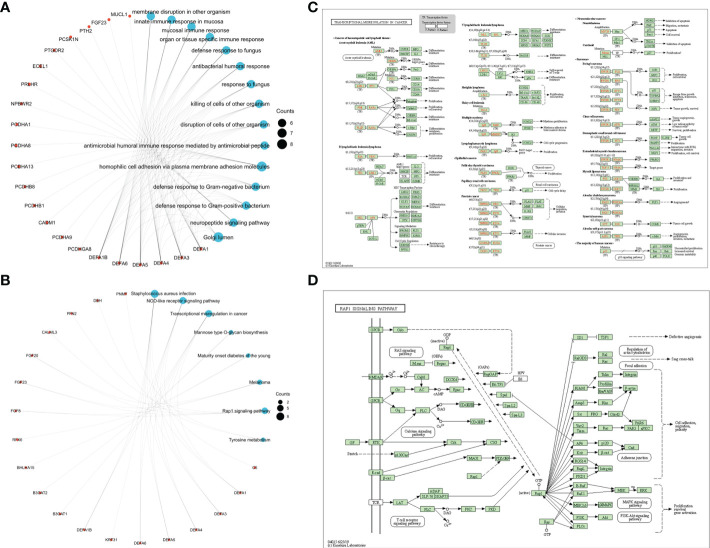
Enrichment Network and pathways of DEFAs. **(A)** Enrichment Network of DEFAs (GO); **(B)** Enrichment Network of DEFAs (KEGG); **(C)** Transcription Disorders Signaling Pathway; **(D)** Rap1 Signaling Pathway.

Kyoto Encyclopedia of Genes and Genomes (KEGG), Gene ontology (GO) and Gene Set Enrichment Analysis (GSEA) were carried to identify and confirm related biological processes. The KEGG and GO enrichment analyses were conducted by R package “org.Hs.eg.db” and “clusterProfiler” and were visualized by “ggplot2”. The functions of DEFAs and the genes significantly related to changes in DEFAs were predicted. The GO enrichment analysis predicts the functional roles of target host genes based on three aspects including biological processes, cellular components, and molecular functions. We found that DEFAs are significantly enriched in GO: 0007156 (homogeneous cell adhesion through plasma membrane adhesion molecules), which is associated with cell proliferation activation and tumor metastasis, and in GO: 0005104 (fibroblast growth factor receptor binding), which is associated with tumor proliferation (**Figure 9A**).

KEGG analysis can identify pathways related to the altered functions of DEFAs and adjacent genes. Eight pathways related to the functions of DEFAs in CRC were enriched in the KEGG analysis (**Figure 9B**). Among these pathways, hsa05202 (**Figure 9C**): transcriptional misregulation in cancer and hsa04015 (**Figure 9D**): Rap1 signaling pathway were probably the key pathways involved in the occurrence and development of CRC.

### Relationship between DEFAs and immune cell infiltration in CRC

**Figure 10:**
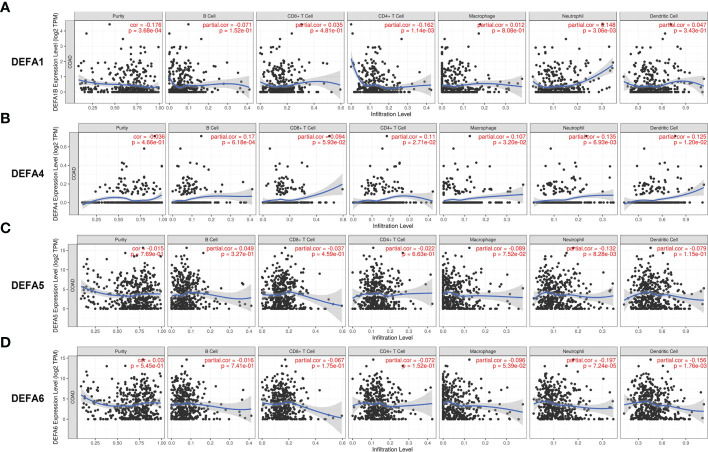
Immune infiltration of DEFAs in CRC. **(A)** Immune infiltration of DEFA1 in CRC; **(B)** Immune infiltration of DEFA4 in CRC; **(C)** Immune infiltration of DEFA5 in CRC; **(D)** Immune infiltration of DEFA6 in CRC.

We analyzed the relationship between DEFAs and immune cell infiltration with the GSVA package of R software (40, 41). The expression of DEFA1 (**Figure 10A**) was negatively correlated with the infiltration of CD4 + T cells (P<0.05, r=-0.162) and positively correlated with the infiltration of neutrophils (P<0.05, r=0.148) and dendritic cells (P<0.05, r=0.047). The expression of DEFA4 (**Figure 10B**) was positively correlated with the infiltration of B cells (P<0.05, r=0.17), CD4+ T cells (P<0.05, r=0.11), macrophages (P<0.05, r=0.107), neutrophils (P<0.05, r=0.135), and dendritic cells (P<0.05, r=0.125). The expression of DEFA5 (**Figure 10C**) was negatively correlated with the infiltration of neutrophils (P<0.05, r=-0.132). The expression of DEFA6 (**Figure 10D**) was negatively correlated with the infiltration of neutrophils (P<0.05, r=-0.197) and dendric cells (P < 0.05, r=-0.156).

## Discussion

The relationship between DEFAs and tumor have been partially reported, and DEFAs are considered to be closely related to CRC.This study focuses on gene expression, related genes, mutation, and enrichment signal of the DEFA family in CRC. Our study showed that DEFA5 and DEFA6 were highly expressed in CRC. The elevation of DEFA5 was related to good prognosis, while that of DEFA6 may be associated with poor prognosis in CRC in M1.Both showed a certain degree of sensitivity and specificity in predicting the prognosis of CRC. Among the co-expressed genes shared by DEFA5 and DEFA6, KLK12, which is responsible for tumor formation, was highly expressed in CRC. The enrichment results showed that the alterations of DEFAs were not only related to cell proliferation and activation, tumor metastasis and tumor proliferation but also to two pathways, namely, Hsa05202: transcriptional misregulation in cancer and hsa04015: Rap1 signaling pathway, which were probably involved in the occurrence and development of CRC.

Previous literature reported that the DEFA1-3 expression levels increased in body fluid levels and mRNA transcription levels in patients with CRC.DEFA1-3 were considered to be potentially important regulators of neovascularization (42). In our present study, we did not find the positive results of the expression of DEFA1-3 in CRC tissues. By using the Oncomine database, we found that the mRNA expression level of DEFAs was up-regulated in different degrees in CRC, and the multiple change in DEFA1-3 in colon cancer changed to 1.171–1.986, which did not show significant difference. This finding may be due to differences in data, tumor stages, and tumor types. Another possible reason is that DEFA is a secretory protein, so it is highly expressed in the serum of patients with colon cancer but not in tumor tissues.

Thus far, limited information is known about the expression and role of DEFA4 in CRC. The TCGA analysis showed that the expression of DEFA4 in CRC tissues was lower than that in normal tissues. The Oncomine database showed that the fold change of DEFA4 in colorectal adenocarcinoma was 1.105. The relationship between DEFA4 and CRC needs to be verified using a larger sample size.

Using the TCGA database, we found that the expression of DEFA5 in tumor tissues was generally lower than that in normal tissues, but the expression in colon cancer and rectal adenocarcinoma cells was significantly higher than that in normal tissues. We obtained the same results using the Oncomine database. Compared with normal tissues, the fold change of DEFA5 in colorectal adenocarcinoma was 15.963 (Skrzypczak Colorectal dataset) (35). The fold changes in colorectal mucinous adenocarcinoma and CRC were 1.135–2.740. Hence, DEFA5 was found to be closely related to colorectal adenocarcinoma. In addition, the high expression of DEFA5 was associated with better prognosis of CRC. Although the **Figure 5**. showed that 5A and 5C as well as 5B and 5D were opposite, while only 5C and 5B were statistically significant. DEFA5 was associated with better survival, whereas DEFA6 was associated with worse survival among patients with CRC in M1. DEFA5 was also found to be highly expressed in colon cancer tissues and showed strong killing effect on colon cancer cells without affecting normal host cells (20). Therefore, we believe that DEFA5 may play an anti-cancer role in the occurrence and development of CRC, consistent with previous results of gastric cancer (13).

Many related studies on DEFA6 and CRC are available. Regardless of contents in blood (23), mRNA expression (24), and protein contents (25), a number of works showed its high expression in CRC. Moreover, DEFA6 had a promoting effect on the proliferation, migration, invasion, and colony formation of CRC cell lines *in vitro*, and the growth rate of cancer cells was significantly decreased by shDEFA6 (25). This is basically consistent with our study. We found that DEFA6 was related to the shorter overall survival rate of patients with CRC with M1 stage, and we thought it might be an independent prognostic marker and good target of CRC. In the Oncomine and TCGA databases, we found the significantly higher expression of DEFA6 in CRC than that in normal tissues, especially in colorectal adenocarcinoma. The expression of the DEFA6 protein in CRC was also increased in the HPA database. The increase inDEFA6 was closely related to the occurrence and development of CRC.

Moreover, DEFA5 and DEFA6 have a certain degree of sensitivity and specificity in predicting CRC. DEFA5 and DEFA6 also showed a mutation rate of 7% in CRC, and their expression was highly correlated. Based on the co-expressed genes of DEFA5 and DEFA6, we found that the co-expressed genes shared by DEFA5 and DEFA6: KLK12, PTGDR2, ATOH1, OLFM4, and CLCA1were involved in tumor formation, inflammatory response, transcriptional regulation, inhibition of cell growth, induction of cell differentiation and apoptosis, and promotion of cell adhesion and tumor inhibition. GO enrichment analysis showed that some signals were affected by DEFAs: GO: 0007156 (homogeneous cell adhesion through plasma membrane adhesion molecules) was related to cell proliferation and activation and tumor metastasis, and GO: 0005104 (fibroblast growth factor receptor binding) was related to tumor proliferation. Eight pathways related to the functions of DEFAs in CRC were found through KEGG. Among these pathways, hsa05202: transcriptional misregulation in cancer and hsa04015: Rap1 signaling pathway were found to be involved in the occurrence and development of CRC. Existing data suggest that DEFA5 and DEFA6 increase concomitantly, but they play different roles. DEFA5 is likely to increase passively with the progression of cancer and has the effect of inhibiting cancer. DEFA6 promotes tumor progression and is associated with poor prognosis.

Defensins, as the innate immune barrier of animals and plants, has a long history of gene evolution, that is, from plants to insects to animals to mammals to primates. Human beings only retain α-defensins that are responsible for the mucosal immunity of the respiratory tract, digestive tract, and urinary system. Their distribution is specific, which indicates that they have particular significance for specific malignant tumors. Paneth cell α-defensins are antimicrobial peptides involved in controlling intestinal microorganisms and immune homeostasis (43).Intestinal symbiotic microorganisms can regulate the expression of DEFAs. The DEFA genes of Paneth cells are regulated by symbiotic bacteria through TLR-MyD88 signal (44). Combined with the bactericidal and antiviral immune properties of defensins, a mature feedback and response mode with intestinal flora are formed to continuously achieve a new balance between DEFAs and intestinal flora. DEFA5 retains the traditional basic immune function. Based on the immune infiltration analysis, the expression of DEFA5 in CRC is negatively correlated with neutrophil infiltration, so DEFA5 may reduce the pressure of the active immunity of neutrophils in the development of CRC. However, DEFA6 loses its natural immune function due to its structural specificity. We also found that their performance was negatively correlated with the infiltration of dendritic cells, which are the most powerful antigen-presenting cells in the human body. This finding may explain that in contrast to DEFA5, DEFA6 is continuously increased during the development of CRC. This also may be the reason why DEFA5 and DEFA6 play opposite roles in the occurrence and development of CRC.

DEFAs showed selective cytotoxicity to human cancer cells usually through cell membrane lysis, triggering cancer cell apoptosis by mitochondrial membrane destruction, or an effective inhibitor of vascular development related to tumor progression (45). DEFAs can activate chemotactic phagocytes and further lead to the destruction of their own normal cells or tumor cells; they can induce target DNA damage and may lead to target cell death (46). Intestinal flora is considered to be closely related to inflammatory response and tumor progression. The release of defensin is one-time, and it can be secreted by neutrophils and is found in tumor cells. Thus, the cytotoxicity of defensin may act on normal and tumor cells based on different concentrations or different mechanisms, so as to promote or inhibit tumor.

This study has some limitations. The results were obtained from tumor-related databases. The final results may be biased due to differences in sources, detection methods, and judgment standards among different samples. In future studies, if we can expand the sample size in the same study, and make the inclusion criteria more strictly and even distinguish tumor pathological types, then we will obtain accurate correlation results between DEFAs and different pathological types of CRC. In addition, if future studies can overexpress or silence DEFA family members *in vivo* and *in vitro* to verify their specific functions on CRC cells and the corresponding tumor microenvironment, evidence will be sufficient to support the existing results.

In conclusion, this study is the first to comprehensively explore mRNA, protein expression and prognostic value of different DEFAs in CRC. We believe that DEFA5 and DEFA6 may be important targets for the pathogenesis, diagnosis, prognosis, and drug screening of CRC. We hope that our findings will help expand existing knowledge, improve treatment design, and increase the accuracy of determining prognosis in patients with CRC.

## Data availability statement

These data were derived from the following resources available in the public domain: Oncomine gene expression array datasets (https://www.oncomine.org/resource/login.html, an online cancer microarray database), GEPIA2 database (http://gepia2.cancer-pku.cn/), cBioPortal (http://www.cbioportal.org/), TIMER (https://cistrome.shinyapps.io/timer/), UCSC XENA (UCSC Xena (xenabrowser.net)) (UCSC Xena (xenabrowser.net)), TCGA (https://www.genome.gov/Funded-Programs-Projects/Cancer-Genome-Atlas), HPA (https://www.proteinatlas.org/), Gene Ontology (http://geneontology.org/), KEGG (https://www.genome.jp/kegg/), STRING (https://cn.string-db.org/), the Gene Expression Omnibus database (https://www.ncbi.nlm.nih.gov/gds) (accession numbers GSE15781).

## Ethics statement

The studies involving human participants were reviewed and approved by Academic Committee of Xi’an Jiaotong University. The patients/participants provided their written informed consent to participate in this study.

## Author contributions

XZ: paper writing; ML: mapping; RS, LL, CD: supervision; MZ, HY, ZD, DW: design of drawing; BM, YY, FY, ZL: data analysis. All authors contributed to the article and approved the submitted version.

## References

[1] Sung H Ferlay J Siegel RL Laversanne M Soerjomataram I Jemal A. Global cancer statistics 2020: GLOBOCAN estimates of incidence and mortality worldwide for 36 cancers in 185 countries. CA Cancer J Clin (2021) 71:209–49. doi: 10.3322/caac.21660 33538338

[2] Song Y Wang L Ran W Li G Xiao Y Wang X. Effect of tumor location on clinicopathological and molecular markers in colorectal cancer in Eastern China patients: An analysis of 2,356 cases. Front Genet (2020) 11:96. doi: 10.3389/fgene.2020.00096 32161617 PMC7052354

[3] Li L Du X Fan G. Identifying potential biomarkers of prognostic value in colorectal cancer *via* tumor microenvironment data mining. Front Genet (2021) 12:787208. doi: 10.3389/fgene.2021.787208 35251116 PMC8890124

[4] Zhao L Lu W. Defensins in innate immunity. Curr Opin Hematol (2014) 21:37–42. doi: 10.1097/MOH.0000000000000005 24275690

[5] Hill CP Yee J Selsted ME Eisenberg D. Crystal structure of defensin HNP-3, an amphiphilic dimer: mechanisms of membrane permeabilization. Science (1991) 251:1481–5. doi: 10.1126/science.2006422 2006422

[6] Ganz T Lehrer RI. Defensins. Pharmacol Ther (1995) 66:191–205. doi: 10.1016/0163-7258(94)00076-F 7667395

[7] Bastian A Schafer H. Human alpha-defensin 1 (HNP-1) inhibits adenoviral infection in vitro. Regul Pept (2001) 101:157–61. doi: 10.1016/S0167-0115(01)00282-8 11495691

[8] Gunes M Gecit I Pirincci N Kemik AS Purisa S Ceylan K. Plasma human neutrophil proteins-1, -2, and -3 levels in patients with bladder cancer. J Cancer Res Clin Oncol (2013) 139:195–9. doi: 10.1007/s00432-012-1305-0 PMC11824696 23011762

[9] Muller CA Markovic-Lipkovski J Klatt T Gamper J Schwarz G Beck H. Human alpha-defensins HNPs-1, -2, and -3 in renal cell carcinoma: influences on tumor cell proliferation. Am J Pathol (2002) 160:1311–24. doi: 10.1016/S0002-9440(10)62558-8 PMC1867209 11943716

[10] Bauer JA Chakravarthy AB Rosenbluth JM Mi D Seeley EH De Matos Granja-Ingram N. Identification of markers of taxane sensitivity using proteomic and genomic analyses of breast tumors from patients receiving neoadjuvant paclitaxel and radiation. Clin Cancer Res (2010) 16:681–90. doi: 10.1158/1078-0432.CCR-09-1091 PMC2892225 20068102

[11] Winter J Pantelis A Kraus D Reckenbeil J Reich R Jepsen S. Human alpha-defensin (DEFA) gene expression helps to characterise benign and malignant salivary gland tumours. BMC Cancer (2012) 12:465. doi: 10.1186/1471-2407-12-465 23050799 PMC3518101

[12] Mohri Y Mohri T Wei W Qi YJ Martin A Miki C. Identification of macrophage migration inhibitory factor and human neutrophil peptides 1-3 as potential biomarkers for gastric cancer. Br J Cancer (2009) 101:295–302. doi: 10.1038/sj.bjc.6605138 19550422 PMC2720195

[13] Wu Z Ding Z Cheng B Cui Z. The inhibitory effect of human DEFA5 in growth of gastric cancer by targeting BMI1. Cancer Sci (2021) 112:1075–83. doi: 10.1111/cas.14827 PMC7935777 33503272

[14] Mothes H Melle C Ernst G Kaufmann R von Eggeling F Settmacher U. Human neutrophil peptides 1-3–early markers in development of colorectal adenomas and carcinomas. Dis Markers (2008) 25:123–9. doi: 10.1155/2008/693937 PMC3827812 18957723

[15] Melle C Ernst G Schimmel B Bleul A Thieme H Kaufmann R. Discovery and identification of alpha-defensins as low abundant, tumor-derived serum markers in colorectal cancer. Gastroenterology (2005) 129:66–73. doi: 10.1053/j.gastro.2005.05.014 16012935

[16] Albrethsen J Bogebo R Gammeltoft S Olsen J Winther B Raskov H. Upregulated expression of human neutrophil peptides 1, 2 and 3 (HNP 1-3) in colon cancer serum and tumours: a biomarker study. BMC Cancer (2005) 5:8. doi: 10.1186/1471-2407-5-8 15656915 PMC548152

[17] Kemik O Kemik AS Sumer A Begenik H Purisa S Tuzun S. Human neutrophil peptides 1, 2 and 3 (HNP 1-3): elevated serum levels in colorectal cancer and novel marker of lymphatic and hepatic metastasis. Hum Exp Toxicol (2013) 32:167–71. doi: 10.1177/0960327111412802 21669914

[18] Bukurova Iu A Nikitina SL Khankin SL Krasnov GS Lisitsin NA Karpov VL. Identification of protein markers for serum diagnosis of cancer based on microRNA expression profiling. Mol Biol (Mosk) (2011) 45:376–81. 21634125

[19] Nastase A Paslaru L Niculescu AM Ionescu M Dumitrascu T Herlea V. Prognostic and predictive potential molecular biomarkers in colon cancer. Chirurgia (Bucur) (2011) 106:177–85. 21696062

[20] Panjeta A Preet S. Anticancer potential of human intestinal defensin 5 against 1, 2-dimethylhydrazine dihydrochloride induced colon cancer: A therapeutic approach. Peptides (2020) 126:170263. doi: 10.1016/j.peptides.2020.170263 31981594

[21] Qiao Q Bai R Song W Gao H Zhang M Lu J. Human alpha-defensin 5 suppressed colon cancer growth by targeting PI3K pathway. Exp Cell Res (2021) 407:112809. doi: 10.1016/j.yexcr.2021.112809 34487729

[22] Szyk A Wu Z Tucker K Yang D Lu W Lubkowski J. Crystal structures of human alpha-defensins HNP4, HD5, and HD6. Protein Sci (2006) 15:2749–60. doi: 10.1110/ps.062336606 PMC2242434 17088326

[23] Nam MJ Kee MK Kuick R Hanash SM. Identification of defensin alpha6 as a potential biomarker in colon adenocarcinoma. J Biol Chem (2005) 280:8260–5. doi: 10.1074/jbc.M410054200 15613481

[24] Radeva MY Jahns F Wilhelm A Glei M Settmacher U Greulich KO. Defensin alpha 6 (DEFA 6) overexpression threshold of over 60 fold can distinguish between adenoma and fully blown colon carcinoma in individual patients. BMC Cancer (2010) 10:588. doi: 10.1186/1471-2407-10-588 20979654 PMC2984430

[25] Jeong D Kim H Kim D Ban S Oh S Ji S. Defensin alpha 6 (DEFA6) is a prognostic marker in colorectal cancer. Cancer Biomark (2019) 24:485–95. doi: 10.3233/CBM-182221 PMC13082530 30932884

[26] Vivian J Rao AA Nothaft FA Ketchum C Armstrong J Novak A. Toil enables reproducible, open source, big biomedical data analyses. Nat Biotechnol (2017) 35:314–6. doi: 10.1038/nbt.3772 PMC5546205 28398314

[27] Davis S Meltzer PS. GEOquery: a bridge between the gene expression omnibus (GEO) and BioConductor. Bioinformatics (2007) 23:1846–7. doi: 10.1093/bioinformatics/btm254 17496320

[28] Smyth GK. Limma: Linear models for microarray data. In: Bioinformatics and computational biology solutions using r and bioconductor. New York: Springer New York (2013). p. 397–420.

[29] Gu Z Eils R Schlesner M. Complex heatmaps reveal patterns and correlations in multidimensional genomic data. Bioinformatics (2016) 32:2847–9. doi: 10.1093/bioinformatics/btw313 27207943

[30] Tang Z Kang B Li C Chen T Zhang Z. GEPIA2: an enhanced web server for large-scale expression profiling and interactive analysis. Nucleic Acids Res (2019) 47:W556–60. doi: 10.1093/nar/gkz430 PMC6602440 31114875

[31] Cancer Genome Atlas N. Comprehensive molecular portraits of human breast tumours. Nature (2012) 490:61–70. doi: 10.1038/nature11412 23000897 PMC3465532

[32] Jensen LJ Kuhn M Stark M Chaffron S Creevey C Muller J. STRING 8–a global view on proteins and their functional interactions in 630 organisms. Nucleic Acids Res (2009) 37:D412–6. doi: 10.1093/nar/gkn760 PMC2686466 18940858

[33] Li T Fan J Wang B Traugh N Chen Q Liu JS. TIMER: A web server for comprehensive analysis of tumor-infiltrating immune cells. Cancer Res (2017) 77:e108–10. doi: 10.1158/1538-7445.AM2017-108 PMC6042652 29092952

[34] Zou TT Selaru FM Xu Y Shustova V Yin J Mori Y. Application of cDNA microarrays to generate a molecular taxonomy capable of distinguishing between colon cancer and normal colon. Oncogene (2002) 21:4855–62. doi: 10.1038/sj.onc.1205613 12101425

[35] Skrzypczak M Goryca K Rubel T Paziewska A Mikula M Jarosz D. Modeling oncogenic signaling in colon tumors by multidirectional analyses of microarray data directed for maximization of analytical reliability. PloS One 5 (2010) 5(10):e13091. doi: 10.1371/journal.pone.0013091 PMC2948500 20957034

[36] Kaiser S Park YK Franklin JL Halberg RB Yu M Jessen WJ. Transcriptional recapitulation and subversion of embryonic colon development by mouse colon tumor models and human colon cancer. Genome Biol (2007) 8:R131. doi: 10.1186/gb-2007-8-7-r131 17615082 PMC2323222

[37] Hong Y Downey T Eu KW Koh PK Cheah PY ‘metastasis-prone’ signature for early-stage mismatch-repair proficient sporadic colorectal cancer patients A. And its implications for possible therapeutics. Clin Exp Metastasis (2010) 27:83–90. doi: 10.1007/s10585-010-9305-4 20143136

[38] Sabates-Bellver J van der Flier LG de Palo M Cattaneo E Maake C Rehrauer H. Transcriptome profile of human colorectal adenomas. Mol Cancer Res (2007) 5:1263–75. doi: 10.1158/1541-7786.MCR-07-0267 18171984

[39] Liu J Lichtenberg T Hoadley KA Poisson LM Lazar AJ Cherniack AD. An integrated TCGA pan-cancer clinical data resource to drive high-quality survival outcome analytics. Cell (2018) 173:400–416.e11. doi: 10.1016/j.cell.2018.02.052 29625055 PMC6066282

[40] Hanzelmann S Castelo R Guinney J. GSVA: Gene set variation analysis for microarray and RNA-seq data. BMC Bioinf (2013) 14:7. doi: 10.1186/1471-2105-14-7 PMC3618321 23323831

[41] Bindea G Mlecnik B Tosolini M Kirilovsky A Waldner M Obenauf AC. Spatiotemporal dynamics of intratumoral immune cells reveal the immune landscape in human cancer. Immunity (2013) 39:782–95. doi: 10.1016/j.immuni.2013.10.003 24138885

[42] Chavakis T Cines DB Rhee JS Liang OD Schubert U Hammes HP. Regulation of neovascularization by human neutrophil peptides (alpha-defensins): A link between inflammation and angiogenesis. FASEB J (2004) 18:1306–8. doi: 10.1096/fj.03-1009fje 15208269

[43] Wehkamp J Stange EF. An update review on the paneth cell as key to ileal crohn’s disease. Front Immunol (2020) 11:646. doi: 10.3389/fimmu.2020.00646 32351509 PMC7174711

[44] Menendez A Willing BP Montero M Wlodarska M So CC Bhinder G. Bacterial stimulation of the TLR-MyD88 pathway modulates the homeostatic expression of ileal paneth cell alpha-defensins. J Innate Immun (2013) 5:39–49. doi: 10.1159/000341630 22986642 PMC6741583

[45] Mader JS Hoskin DW. Cationic antimicrobial peptides as novel cytotoxic agents for cancer treatment. Expert Opin Investig Drugs (2006) 15:933–46. doi: 10.1517/13543784.15.8.933 16859395

[46] Gera JF Lichtenstein A. Human neutrophil peptide defensins induce single strand DNA breaks in target cells. Cell Immunol (1991) 138:108–20. doi: 10.1016/0008-8749(91)90136-Y 1913832

