# Supplementary material for: Comprehensive analysis of alfa defensin expression and prognosis in human colorectal cancer
Source: Front Oncol. 2023 Jan 10;12:974654. doi: 10.3389/fonc.2022.974654 (PMC9872005; doi:10.3389/fonc.2022.974654)

**Supplementary Table S1. Basic information of colon cancer patients for immunohistochemistry**

| **number** | **age** | **sex** | **Pathological results** |
| --- | --- | --- | --- |
| 1 | 74 | Male | "Sigmoid colon" ulcerative adenocarcinoma grade II, infiltrating the lower serosa layer of intestinal wall; Tubular adenoma with intraepithelial neoplasia becomes high grade; No cancer infiltration was found at the upper and lower surgical margins; Periintestinal lymph node 0/16 cancers metastasized. Immunohistochemical results: TS (-), TOPO-II (Grade I), Pgp (2+), GST - π (2+), LRP (3+), ERCC-1 (-), Braf (-), MLH1 (+), MSH6 (+), PMS2 (+), MSH2 (+), Ber EP4 (focus+), Ki67 (70%). |
| 2 | 84 | Female | (Ascending colon) Ulcerative adenocarcinoma grade II, serous layer leached, tumor thrombus can be seen in the vessels; Tubular adenoma; No cancer infiltration was found at the upper and lower surgical margins; Periintestinal lymph node 5/19 cancer metastasis; Chronic appendicitis. AJCC Version 8 TNM staging: pTNM T4aN2aM0 Immunohistochemical results: TS (1+), TOPO-II (Grade I), Pgp (2+), GST - π (2+), LRP (2+), ERCC-1 (-), Braf (-), MLH1 (+), MSH6 (+), PMS2 (+), MSH2 (+), Ber-EP4 (-), Ki67 (80%). |
| 3 | 64 | Female | (Sigmoid colon) ulcerative adenocarcinoma grade II, infiltrating the subserous adipose tissue of intestinal wall; Tubular villous adenoma with grade ⅱ intraepithelial neoplasia; No cancer infiltration was found at the upper and lower circumferential incisional margins; 1/18 periintestinal lymph nodes showed cancer metastasis. Immunohistochemical results: Syn (-), CgA (-), Ki67 (60%), MSH2 (+), MSH6 (+), MLH1 (+), PMS2 (+), BER-EP4 (+). |
| 4 | 68 | Female | Grade II of "sigmoid colon" protuberant adenocarcinoma with necrosis, lymphocyte and histocyte reaction, infiltrating into the serosa; Tubular adenoma with low grade intraepithelial tumor (2); No cancer infiltration was found at the upper and lower surgical margins; 5/32 periintestinal lymph nodes metastasized; "Descending colon" tubular adenoma with intraepithelial tumor becomes lower grade. Immunohistochemical results: TS (-), TOPO-II (Grade II), Pgp (2+), GST - π (1+), LRP (2+), ERCC-1 (1+), Braf (-), Ki67 (80%). |
| 5 | 65 | Male | Ulcerative adenocarcinoma grade II - III and signet ring cell carcinoma of the right colon infiltrate the whole layer of the intestinal wall to the serosa, and many tumor emboli can be seen in the vessels; No cancer infiltration was found at the upper, lower and radial surgical margins; Mesenteric lymph node 3/24 cancer metastasis; Chronic appendicitis; No cancer infiltration was found in omental tissue; Tubular adenoma of colon. Immunohistochemical results: TS (-), TOPO-II (-), Pgp (2+), GST - π (1+), LRP (2+), ERCC-1 (-), Braf (-), Ki67 (80%) Mismatch repair protein detection: MLH1 (+), MSH6 (+), PMS2 (+), MSH2 (+), Ber EP4 (+) interpreted as pMMR |

**Supplementary Table S2. Related frequently altered neighbor genes of DEFA5 in CRC**

| gene_name | gene_id | gene_biotype | cor_pearson | p_pearson | cor_spearman | p_spearman |
| --- | --- | --- | --- | --- | --- | --- |
| DEFA6 | ENSG00000164822 | protein_coding | 0.87304951 | 3.3773E-151 | 0.8599523 | 9.9563E-142 |
| KLK12 | ENSG00000186474 | protein_coding | 0.57254711 | 3.74043E-43 | 0.54830774 | 5.06033E-39 |
| CLCA1 | ENSG00000016490 | protein_coding | 0.49237626 | 1.11779E-30 | 0.52757517 | 9.53978E-36 |
| L1TD1 | ENSG00000240563 | protein_coding | 0.47065632 | 7.84629E-28 | 0.50234577 | 4.69982E-32 |
| REG3A | ENSG00000172016 | protein_coding | 0.45658389 | 4.295E-26 | 0.47098384 | 7.13245E-28 |
| DEFA8P | ENSG00000223629 | unprocessed_pseudogene | 0.44879816 | 3.63549E-25 | 0.41523577 | 1.97678E-21 |
| HEPACAM2 | ENSG00000188175 | protein_coding | 0.43756899 | 7.19488E-24 | 0.46524697 | 3.73608E-27 |
| ITLN2 | ENSG00000158764 | protein_coding | 0.43473109 | 1.50348E-23 | 0.45205701 | 1.49693E-25 |
| OLFM4 | ENSG00000102837 | protein_coding | 0.4343666 | 1.65192E-23 | 0.45027148 | 2.43701E-25 |
| RETNLB | ENSG00000163515 | protein_coding | 0.4326094 | 2.59676E-23 | 0.44123834 | 2.74584E-24 |
| PTGDR2 | ENSG00000183134 | protein_coding | 0.42754355 | 9.42689E-23 | 0.40309416 | 3.52606E-20 |
| ATOH1 | ENSG00000172238 | protein_coding | 0.4245671 | 1.99057E-22 | 0.43175374 | 3.23351E-23 |
| SPINK4 | ENSG00000122711 | protein_coding | 0.41819472 | 9.62018E-22 | 0.42999172 | 5.06929E-23 |
| ELAPOR1 | ENSG00000116299 | protein_coding | 0.41031326 | 6.44843E-21 | 0.38769105 | 1.15476E-18 |
| DEFA9P | ENSG00000233238 | unprocessed_pseudogene | 0.40749851 | 1.25683E-20 | 0.35254077 | 1.71135E-15 |
| SLC18A1 | ENSG00000036565 | protein_coding | 0.39807653 | 1.12102E-19 | 0.39902681 | 9.01851E-20 |
| B3GNT6 | ENSG00000198488 | protein_coding | 0.38580785 | 1.74719E-18 | 0.42766227 | 9.14858E-23 |
| KLK3 | ENSG00000142515 | protein_coding | 0.38438858 | 2.38295E-18 | 0.41569013 | 1.77065E-21 |
| CACNA2D2 | ENSG00000007402 | protein_coding | 0.38225647 | 3.7875E-18 | 0.35699002 | 7.13195E-16 |
| AC090152.1 | ENSG00000167912 | lncRNA | 0.38193582 | 4.05965E-18 | 0.35325932 | 1.48715E-15 |
| NEURL1 | ENSG00000107954 | protein_coding | 0.37597811 | 1.4535E-17 | 0.3981564 | 1.10074E-19 |
| LRRC26 | ENSG00000184709 | protein_coding | 0.37458965 | 1.9493E-17 | 0.39750518 | 1.27724E-19 |
| ATOH8 | ENSG00000168874 | protein_coding | 0.37428069 | 2.08046E-17 | 0.36808136 | 7.57235E-17 |
| GUCA2A | ENSG00000197273 | protein_coding | 0.368578 | 6.83482E-17 | 0.42148951 | 4.278E-22 |
| ITLN1 | ENSG00000179914 | protein_coding | 0.36443754 | 1.59735E-16 | 0.40229628 | 4.24365E-20 |
| PCSK1 | ENSG00000175426 | protein_coding | 0.35980761 | 4.06789E-16 | 0.38059201 | 5.42535E-18 |
| VWA5B2 | ENSG00000145198 | protein_coding | 0.35875826 | 5.01724E-16 | 0.24348101 | 6.59022E-08 |
| AL162739.1 | ENSG00000237227 | processed_pseudogene | 0.35844515 | 5.34049E-16 | 0.35939758 | 4.41575E-16 |
| FCGBP | ENSG00000275395 | protein_coding | 0.35518186 | 1.01957E-15 | 0.34947373 | 3.10387E-15 |
| SERPINA11 | ENSG00000186910 | protein_coding | 0.35200399 | 1.90018E-15 | 0.36091447 | 3.25774E-16 |
| FZD9 | ENSG00000188763 | protein_coding | 0.3509105 | 2.35033E-15 | 0.31233594 | 2.54142E-12 |
| AC011523.1 | ENSG00000267968 | lncRNA | 0.35051525 | 2.53755E-15 | 0.33332166 | 6.42142E-14 |
| PRSS2 | ENSG00000275896 | protein_coding | 0.34936808 | 3.16781E-15 | 0.39391073 | 2.88542E-19 |
| RASD1 | ENSG00000108551 | protein_coding | 0.3484924 | 3.75006E-15 | 0.35970219 | 4.15467E-16 |
| KIF19 | ENSG00000196169 | protein_coding | 0.34583772 | 6.23437E-15 | 0.38766108 | 1.16242E-18 |
| KLK15 | ENSG00000174562 | protein_coding | 0.34571575 | 6.38094E-15 | 0.37540616 | 1.64058E-17 |
| ANO7 | ENSG00000146205 | protein_coding | 0.34407453 | 8.71478E-15 | 0.36001367 | 3.90339E-16 |
| RASD2 | ENSG00000100302 | protein_coding | 0.34149661 | 1.41672E-14 | 0.35938268 | 4.42893E-16 |
| SH2D6 | ENSG00000152292 | protein_coding | 0.33940859 | 2.09305E-14 | 0.33285428 | 6.99149E-14 |
| PLA2G4D | ENSG00000159337 | protein_coding | 0.33900235 | 2.25739E-14 | 0.2799139 | 4.32507E-10 |
| SLITRK6 | ENSG00000184564 | protein_coding | 0.33333554 | 6.4052E-14 | 0.3852982 | 1.95349E-18 |
| MB | ENSG00000198125 | protein_coding | 0.3319657 | 8.21532E-14 | 0.33707293 | 3.22748E-14 |
| MISP3 | ENSG00000141854 | protein_coding | 0.32649692 | 2.19177E-13 | 0.31301798 | 2.26516E-12 |
| TOX | ENSG00000198846 | protein_coding | 0.32409372 | 3.35257E-13 | 0.31435225 | 1.80699E-12 |
| FOXA3 | ENSG00000170608 | protein_coding | 0.32220265 | 4.67172E-13 | 0.32375974 | 3.5555E-13 |
| IGFBP2 | ENSG00000115457 | protein_coding | 0.32012 | 6.71444E-13 | 0.33337862 | 6.35514E-14 |
| EPHB3 | ENSG00000182580 | protein_coding | 0.31870177 | 8.58201E-13 | 0.3111879 | 3.08258E-12 |
| REG1A | ENSG00000115386 | protein_coding | 0.31724386 | 1.10297E-12 | 0.33750782 | 2.97827E-14 |
| DEFA7P | ENSG00000206042 | unprocessed_pseudogene | 0.31316905 | 2.20806E-12 | 0.19700208 | 1.37684E-05 |
| REP15 | ENSG00000174236 | protein_coding | 0.31303385 | 2.25909E-12 | 0.32485061 | 2.93373E-13 |
| MYCN | ENSG00000134323 | protein_coding | 0.31270215 | 2.38923E-12 | 0.30010698 | 1.90365E-11 |
| SP5 | ENSG00000204335 | protein_coding | 0.31199876 | 2.68991E-12 | 0.31395707 | 1.9323E-12 |
| AC244157.2 | ENSG00000275772 | processed_pseudogene | 0.31180421 | 2.77941E-12 | 0.28484219 | 2.06462E-10 |
| SPINK1 | ENSG00000164266 | protein_coding | 0.3114934 | 2.92846E-12 | 0.31743267 | 1.06779E-12 |
| CA8 | ENSG00000178538 | protein_coding | 0.31131036 | 3.01987E-12 | 0.29158407 | 7.33193E-11 |
| DLL1 | ENSG00000198719 | protein_coding | 0.30962317 | 4.00493E-12 | 0.2950853 | 4.23627E-11 |
| FAM222A | ENSG00000139438 | protein_coding | 0.3087997 | 4.5936E-12 | 0.30237805 | 1.31902E-11 |
| SHC2 | ENSG00000129946 | protein_coding | 0.30617388 | 7.09293E-12 | 0.27423655 | 9.95799E-10 |
| BEST3 | ENSG00000127325 | protein_coding | 0.30569141 | 7.67872E-12 | 0.21089214 | 3.14844E-06 |
| OPRD1 | ENSG00000116329 | protein_coding | 0.30067168 | 1.73819E-11 | 0.27595604 | 7.75094E-10 |
| SERPINA1 | ENSG00000197249 | protein_coding | 0.29958118 | 2.07147E-11 | 0.29033525 | 8.90033E-11 |
| COL4A5 | ENSG00000188153 | protein_coding | 0.29566103 | 3.8681E-11 | 0.31429145 | 1.82574E-12 |
| DPEP1 | ENSG00000015413 | protein_coding | 0.29523103 | 4.13997E-11 | 0.31362692 | 2.04347E-12 |
| AC124067.2 | ENSG00000253414 | lncRNA | 0.29521398 | 4.15112E-11 | 0.29403179 | 5.00046E-11 |
| SH2D7 | ENSG00000183476 | protein_coding | 0.29504218 | 4.26519E-11 | 0.31025261 | 3.60534E-12 |
| CD5 | ENSG00000110448 | protein_coding | 0.29412007 | 4.93158E-11 | 0.26929337 | 2.02672E-09 |
| HES6 | ENSG00000144485 | protein_coding | 0.29408851 | 4.9561E-11 | 0.29702188 | 3.11754E-11 |
| DKK4 | ENSG00000104371 | protein_coding | 0.29400272 | 5.02336E-11 | 0.24727736 | 4.04401E-08 |
| CCL24 | ENSG00000106178 | protein_coding | 0.2939013 | 5.104E-11 | 0.29733683 | 2.96523E-11 |
| CBLIF | ENSG00000134812 | protein_coding | 0.29367874 | 5.28545E-11 | 0.3104778 | 3.47206E-12 |
| HMGB1P49 | ENSG00000230519 | processed_pseudogene | 0.29302984 | 5.85111E-11 | 0.25828755 | 9.37008E-09 |
| PTCHD4 | ENSG00000244694 | protein_coding | 0.29272823 | 6.13372E-11 | 0.26608351 | 3.19061E-09 |
| WFDC2 | ENSG00000101443 | protein_coding | 0.29234445 | 6.51263E-11 | 0.28012839 | 4.18935E-10 |
| NKX2-2 | ENSG00000125820 | protein_coding | 0.29156677 | 7.3517E-11 | 0.37321502 | 2.60298E-17 |
| LINC01101 | ENSG00000280409 | TEC | 0.29131314 | 7.64747E-11 | 0.26417751 | 4.16551E-09 |
| GALNT8 | ENSG00000130035 | protein_coding | 0.2907177 | 8.38817E-11 | 0.30432126 | 9.61197E-12 |
| LINC00261 | ENSG00000259974 | lncRNA | 0.28770878 | 1.3339E-10 | 0.29367221 | 5.29086E-11 |
| RAB26 | ENSG00000167964 | protein_coding | 0.28761132 | 1.35397E-10 | 0.29453683 | 4.61869E-11 |
| CRYM | ENSG00000103316 | protein_coding | 0.28756522 | 1.36357E-10 | 0.27973049 | 4.4445E-10 |
| ZG16 | ENSG00000174992 | protein_coding | 0.28676129 | 1.54194E-10 | 0.36328082 | 2.02043E-16 |
| TPSG1 | ENSG00000116176 | protein_coding | 0.28631238 | 1.65123E-10 | 0.32668347 | 2.1203E-13 |
| AC093866.1 | ENSG00000251095 | lncRNA | 0.28450636 | 2.17233E-10 | 0.24217611 | 7.78022E-08 |
| AXIN2 | ENSG00000168646 | protein_coding | 0.28426098 | 2.25446E-10 | 0.2773628 | 6.30614E-10 |
| NCKAP5 | ENSG00000176771 | protein_coding | 0.28332101 | 2.59802E-10 | 0.32235443 | 4.54934E-13 |
| PLA2G4F | ENSG00000168907 | protein_coding | 0.28281435 | 2.80381E-10 | 0.27372554 | 1.07242E-09 |
| FGF20 | ENSG00000078579 | protein_coding | 0.282775 | 2.82044E-10 | 0.26223304 | 5.45582E-09 |
| NPW | ENSG00000183971 | protein_coding | 0.28188616 | 3.22275E-10 | 0.28066217 | 3.86935E-10 |
| CCDC60 | ENSG00000183273 | protein_coding | 0.28174518 | 3.2915E-10 | 0.30667163 | 6.5344E-12 |
| CALML3 | ENSG00000178363 | protein_coding | 0.27969467 | 4.4682E-10 | 0.28293926 | 2.75165E-10 |
| WNK2 | ENSG00000165238 | protein_coding | 0.27901586 | 4.94123E-10 | 0.28934774 | 1.03678E-10 |
| SMAD9 | ENSG00000120693 | protein_coding | 0.27712958 | 6.52607E-10 | 0.27545628 | 8.33796E-10 |
| UGT2B7 | ENSG00000171234 | protein_coding | 0.27673713 | 6.9131E-10 | 0.31618138 | 1.32315E-12 |
| HTR3E | ENSG00000186038 | protein_coding | 0.27664521 | 7.00693E-10 | 0.31035832 | 3.54216E-12 |
| EPHB2 | ENSG00000133216 | protein_coding | 0.27658897 | 7.06494E-10 | 0.26458511 | 3.93534E-09 |
| PHGR1 | ENSG00000233041 | protein_coding | 0.27487669 | 9.07297E-10 | 0.29338234 | 5.53687E-11 |
| MSI1 | ENSG00000135097 | protein_coding | 0.27437243 | 9.7634E-10 | 0.26863669 | 2.22497E-09 |
| SERPINA7 | ENSG00000123561 | protein_coding | 0.27433345 | 9.81884E-10 | 0.34425271 | 8.42554E-15 |
| TFF3 | ENSG00000160180 | protein_coding | 0.27365801 | 1.08297E-09 | 0.26522608 | 3.59814E-09 |
| MYCL | ENSG00000116990 | protein_coding | 0.2732801 | 1.14386E-09 | 0.24426074 | 5.9652E-08 |
| WNT4 | ENSG00000162552 | protein_coding | 0.27301399 | 1.18872E-09 | 0.2736807 | 1.07941E-09 |

**Supplementary Table S3. Related frequently altered neighbor genes of DEFA6 in CRC**

| gene_name | gene_id | gene_biotype | cor_pearson | p_pearson | cor_spearman | p_spearman |
| --- | --- | --- | --- | --- | --- | --- |
| DEFA5 | ENSG00000164816 | protein_coding | 0.87304951 | 3.3773E-151 | 0.8599523 | 9.9563E-142 |
| KLK12 | ENSG00000186474 | protein_coding | 0.57101399 | 6.98944E-43 | 0.56128332 | 3.42779E-41 |
| OLFM4 | ENSG00000102837 | protein_coding | 0.45079988 | 2.11034E-25 | 0.46313921 | 6.81158E-27 |
| DEFA8P | ENSG00000223629 | unprocessed_pseudogene | 0.44383104 | 1.38037E-24 | 0.43725282 | 7.81326E-24 |
| CLCA1 | ENSG00000016490 | protein_coding | 0.43086056 | 4.06258E-23 | 0.45936054 | 1.9785E-26 |
| PTGDR2 | ENSG00000183134 | protein_coding | 0.43039015 | 4.58029E-23 | 0.40582857 | 1.86178E-20 |
| DEFA9P | ENSG00000233238 | unprocessed_pseudogene | 0.43009117 | 4.94262E-23 | 0.41913333 | 7.64394E-22 |
| GUCA2A | ENSG00000197273 | protein_coding | 0.42929215 | 6.05563E-23 | 0.4583385 | 2.63392E-26 |
| ATOH1 | ENSG00000172238 | protein_coding | 0.41253699 | 3.78909E-21 | 0.42679722 | 1.1378E-22 |
| HEPACAM2 | ENSG00000188175 | protein_coding | 0.39954436 | 8.00848E-20 | 0.43047699 | 4.48004E-23 |
| ELAPOR1 | ENSG00000116299 | protein_coding | 0.39858751 | 9.97354E-20 | 0.38155984 | 4.40335E-18 |
| REG3A | ENSG00000172016 | protein_coding | 0.39808561 | 1.1187E-19 | 0.40351032 | 3.20068E-20 |
| RETNLB | ENSG00000163515 | protein_coding | 0.39577267 | 1.89412E-19 | 0.42064924 | 5.2646E-22 |
| PLA2G4F | ENSG00000168907 | protein_coding | 0.39184049 | 4.59306E-19 | 0.36790948 | 7.84538E-17 |
| L1TD1 | ENSG00000240563 | protein_coding | 0.38409467 | 2.54064E-18 | 0.40864854 | 9.57627E-21 |
| SERPINA7 | ENSG00000123561 | protein_coding | 0.37954133 | 6.7997E-18 | 0.42476949 | 1.89237E-22 |
| EPHB2 | ENSG00000133216 | protein_coding | 0.37166688 | 3.59914E-17 | 0.34857764 | 3.68906E-15 |
| AXIN2 | ENSG00000168646 | protein_coding | 0.36726548 | 8.95722E-17 | 0.35742245 | 6.54549E-16 |
| CACNA2D2 | ENSG00000007402 | protein_coding | 0.36723833 | 9.00735E-17 | 0.37839608 | 8.68909E-18 |
| AC124067.2 | ENSG00000253414 | lncRNA | 0.36645959 | 1.05686E-16 | 0.37025507 | 4.8292E-17 |
| SPINK4 | ENSG00000122711 | protein_coding | 0.3637275 | 1.84539E-16 | 0.37779227 | 9.88433E-18 |
| DPEP1 | ENSG00000015413 | protein_coding | 0.36241924 | 2.40538E-16 | 0.36829611 | 7.2443E-17 |
| SPINK1 | ENSG00000164266 | protein_coding | 0.36198092 | 2.62801E-16 | 0.3670773 | 9.3104E-17 |
| NEURL1 | ENSG00000107954 | protein_coding | 0.34913129 | 3.31587E-15 | 0.37835082 | 8.77352E-18 |
| WFDC2 | ENSG00000101443 | protein_coding | 0.34886486 | 3.49059E-15 | 0.34748235 | 4.55279E-15 |
| AL121832.1 | ENSG00000233017 | lncRNA | 0.34808711 | 4.05394E-15 | 0.35352381 | 1.4121E-15 |
| HES6 | ENSG00000144485 | protein_coding | 0.34710026 | 4.89852E-15 | 0.35032363 | 2.63352E-15 |
| ITLN2 | ENSG00000158764 | protein_coding | 0.34705605 | 4.94016E-15 | 0.39291228 | 3.61208E-19 |
| MISP3 | ENSG00000141854 | protein_coding | 0.34427532 | 8.38952E-15 | 0.32491424 | 2.90095E-13 |
| RASD2 | ENSG00000100302 | protein_coding | 0.33995501 | 1.89037E-14 | 0.35984476 | 4.03774E-16 |
| CCL24 | ENSG00000106178 | protein_coding | 0.33959235 | 2.02261E-14 | 0.3442018 | 8.50721E-15 |
| VIL1 | ENSG00000127831 | protein_coding | 0.3383862 | 2.53108E-14 | 0.30978729 | 3.89676E-12 |
| LINC01101 | ENSG00000280409 | TEC | 0.33757827 | 2.93971E-14 | 0.31689389 | 1.17121E-12 |
| KLK15 | ENSG00000174562 | protein_coding | 0.33667715 | 3.47199E-14 | 0.37357186 | 2.41505E-17 |
| SLC39A5 | ENSG00000139540 | protein_coding | 0.33605217 | 3.89552E-14 | 0.30169337 | 1.47379E-11 |
| FOXA3 | ENSG00000170608 | protein_coding | 0.33533103 | 4.44737E-14 | 0.3255593 | 2.58825E-13 |
| ATOH8 | ENSG00000168874 | protein_coding | 0.33468391 | 5.00734E-14 | 0.3607145 | 3.39132E-16 |
| APCDD1 | ENSG00000154856 | protein_coding | 0.33401024 | 5.66362E-14 | 0.3329311 | 6.8945E-14 |
| NCKAP5 | ENSG00000176771 | protein_coding | 0.333614 | 6.08827E-14 | 0.33303026 | 6.77125E-14 |
| PCSK1 | ENSG00000175426 | protein_coding | 0.32779474 | 1.73953E-13 | 0.35415898 | 1.24674E-15 |
| FAM222A | ENSG00000139438 | protein_coding | 0.32625785 | 2.28681E-13 | 0.31936659 | 7.65065E-13 |
| MYCN | ENSG00000134323 | protein_coding | 0.32486099 | 2.92836E-13 | 0.31253294 | 2.45841E-12 |
| DEFB1 | ENSG00000164825 | protein_coding | 0.32412104 | 3.33648E-13 | 0.31557315 | 1.46797E-12 |
| ITLN1 | ENSG00000179914 | protein_coding | 0.32321041 | 3.91572E-13 | 0.35422742 | 1.2301E-15 |
| AGT | ENSG00000135744 | protein_coding | 0.32198704 | 4.85112E-13 | 0.34231568 | 1.21464E-14 |
| SLC18A1 | ENSG00000036565 | protein_coding | 0.32178448 | 5.0258E-13 | 0.3518371 | 1.96296E-15 |
| H2AJ | ENSG00000246705 | protein_coding | 0.32131125 | 5.45824E-13 | 0.33895557 | 2.27711E-14 |
| TGM3 | ENSG00000125780 | protein_coding | 0.32108153 | 5.68107E-13 | 0.3439015 | 9.00495E-15 |
| TTC38 | ENSG00000075234 | protein_coding | 0.32087128 | 5.89282E-13 | 0.299001 | 2.27341E-11 |
| IGFBP2 | ENSG00000115457 | protein_coding | 0.32069255 | 6.07887E-13 | 0.32927543 | 1.33457E-13 |
| AGXT | ENSG00000172482 | protein_coding | 0.31959041 | 7.35994E-13 | 0.32642157 | 2.2213E-13 |
| REPIN1 | ENSG00000214022 | protein_coding | 0.31921715 | 7.85098E-13 | 0.31049335 | 3.46304E-12 |
| MB | ENSG00000198125 | protein_coding | 0.31909145 | 8.02344E-13 | 0.34264044 | 1.14259E-14 |
| WNK2 | ENSG00000165238 | protein_coding | 0.31891864 | 8.26661E-13 | 0.3136176 | 2.0467E-12 |
| ALDH4A1 | ENSG00000159423 | protein_coding | 0.31755616 | 1.04537E-12 | 0.30447398 | 9.37492E-12 |
| EMID1 | ENSG00000186998 | protein_coding | 0.31627839 | 1.30138E-12 | 0.31366373 | 2.03077E-12 |
| SLC2A8 | ENSG00000136856 | protein_coding | 0.31623117 | 1.31193E-12 | 0.30468965 | 9.04986E-12 |
| LINC01996 | ENSG00000261863 | lncRNA | 0.31464903 | 1.71814E-12 | 0.32978478 | 1.21788E-13 |
| B3GNT6 | ENSG00000198488 | protein_coding | 0.30945432 | 4.11929E-12 | 0.36242091 | 2.40457E-16 |
| DAPL1 | ENSG00000163331 | protein_coding | 0.30853323 | 4.80159E-12 | 0.36013722 | 3.8079E-16 |
| C1orf53 | ENSG00000203724 | protein_coding | 0.30763325 | 5.5744E-12 | 0.28699276 | 1.48838E-10 |
| B3GNT8 | ENSG00000177191 | protein_coding | 0.3073951 | 5.79845E-12 | 0.29857422 | 2.4341E-11 |
| EPHB3 | ENSG00000182580 | protein_coding | 0.30731774 | 5.8731E-12 | 0.28731556 | 1.41669E-10 |
| HUNK | ENSG00000142149 | protein_coding | 0.30706831 | 6.12026E-12 | 0.30895575 | 4.4759E-12 |
| LINC01605 | ENSG00000253161 | lncRNA | 0.30697887 | 6.21133E-12 | 0.31497262 | 1.62613E-12 |
| GJB1 | ENSG00000169562 | protein_coding | 0.30667918 | 6.52627E-12 | 0.28436161 | 2.22042E-10 |
| UGT2A3 | ENSG00000135220 | protein_coding | 0.30628753 | 6.96144E-12 | 0.30987048 | 3.84302E-12 |
| WNT4 | ENSG00000162552 | protein_coding | 0.30598934 | 7.31165E-12 | 0.30435643 | 9.55687E-12 |
| BEST3 | ENSG00000127325 | protein_coding | 0.30550599 | 7.91619E-12 | 0.2298109 | 3.5795E-07 |
| ILVBL | ENSG00000105135 | protein_coding | 0.30505868 | 8.51898E-12 | 0.31592323 | 1.38282E-12 |
| LRRC26 | ENSG00000184709 | protein_coding | 0.30467509 | 9.07145E-12 | 0.3330327 | 6.76824E-14 |
| EPHX2 | ENSG00000120915 | protein_coding | 0.30359772 | 1.0817E-11 | 0.27950635 | 4.59482E-10 |
| UGT2B7 | ENSG00000171234 | protein_coding | 0.30315015 | 1.1635E-11 | 0.32424278 | 3.2657E-13 |
| HADH | ENSG00000138796 | protein_coding | 0.3019666 | 1.41001E-11 | 0.28675907 | 1.54247E-10 |
| NOTUM | ENSG00000185269 | protein_coding | 0.30189709 | 1.42597E-11 | 0.32001404 | 6.83899E-13 |
| LINC00526 | ENSG00000264575 | lncRNA | 0.30184016 | 1.43918E-11 | 0.27696333 | 6.68737E-10 |
| C10orf99 | ENSG00000188373 | protein_coding | 0.29969619 | 2.03357E-11 | 0.31484194 | 1.66269E-12 |
| KLK3 | ENSG00000142515 | protein_coding | 0.29940311 | 2.13151E-11 | 0.35857574 | 5.20326E-16 |
| LINC02563 | ENSG00000263520 | lncRNA | 0.29877305 | 2.35791E-11 | 0.30282367 | 1.22694E-11 |
| SMAGP | ENSG00000170545 | protein_coding | 0.29867689 | 2.39446E-11 | 0.2869704 | 1.49347E-10 |
| PHPT1 | ENSG00000054148 | protein_coding | 0.29836428 | 2.51716E-11 | 0.29073342 | 8.36775E-11 |
| MSI1 | ENSG00000135097 | protein_coding | 0.29821293 | 2.57875E-11 | 0.28836174 | 1.20673E-10 |
| NUDT14 | ENSG00000183828 | protein_coding | 0.29695465 | 3.15102E-11 | 0.28068749 | 3.85477E-10 |
| MYCL | ENSG00000116990 | protein_coding | 0.29637928 | 3.45233E-11 | 0.28259065 | 2.89965E-10 |
| LGALS4 | ENSG00000171747 | protein_coding | 0.29634045 | 3.47365E-11 | 0.263002 | 4.90488E-09 |
| FZD9 | ENSG00000188763 | protein_coding | 0.2956364 | 3.88319E-11 | 0.29759726 | 2.8448E-11 |
| PMFBP1 | ENSG00000118557 | protein_coding | 0.29517119 | 4.17925E-11 | 0.2970059 | 3.12546E-11 |
| VWA5B2 | ENSG00000145198 | protein_coding | 0.2945806 | 4.58697E-11 | 0.19729485 | 1.33606E-05 |
| ME3 | ENSG00000151376 | protein_coding | 0.29448886 | 4.65369E-11 | 0.28493211 | 2.03667E-10 |
| SELENBP1 | ENSG00000143416 | protein_coding | 0.294295 | 4.79781E-11 | 0.31171281 | 2.82246E-12 |
| SERPINA11 | ENSG00000186910 | protein_coding | 0.29373064 | 5.24258E-11 | 0.30338298 | 1.12022E-11 |
| DACH1 | ENSG00000276644 | protein_coding | 0.29340449 | 5.51769E-11 | 0.29072701 | 8.37606E-11 |
| PRSS2 | ENSG00000275896 | protein_coding | 0.29337112 | 5.54662E-11 | 0.34413931 | 8.6085E-15 |
| FGF20 | ENSG00000078579 | protein_coding | 0.29204557 | 6.82343E-11 | 0.26232582 | 5.38628E-09 |
| GADD45G | ENSG00000130222 | protein_coding | 0.29194704 | 6.92902E-11 | 0.26130065 | 6.20466E-09 |
| NECTIN1 | ENSG00000110400 | protein_coding | 0.29113604 | 7.86084E-11 | 0.28757302 | 1.36194E-10 |
| AC011523.1 | ENSG00000267968 | lncRNA | 0.29108708 | 7.92085E-11 | 0.30196049 | 1.41141E-11 |
| TG | ENSG00000042832 | protein_coding | 0.29096769 | 8.06905E-11 | 0.32879882 | 1.45363E-13 |
| GAL3ST2 | ENSG00000154252 | protein_coding | 0.29014075 | 9.17235E-11 | 0.29847034 | 2.47486E-11 |
| TSPEAR | ENSG00000175894 | protein_coding | 0.2896893 | 9.83537E-11 | 0.24711374 | 4.13071E-08 |

**Supplementary Figure S1. Mutation rates of DEFA1 and 3-6 in CRC**


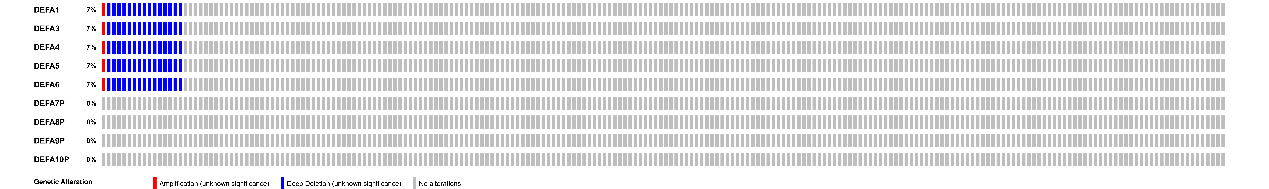

Supplement: Supplementary file 1 [file DataSheet_1.docx]
